# Impact of whole‐body versus nose‐only inhalation exposure systems on systemic, respiratory, and cardiovascular endpoints in a 2‐month cigarette smoke exposure study in the ApoE^−/−^ mouse model

**DOI:** 10.1002/jat.4149

**Published:** 2021-04-06

**Authors:** Ulrike Kogel, Ee Tsin Wong, Justyna Szostak, Wei Teck Tan, Francesco Lucci, Patrice Leroy, Bjoern Titz, Yang Xiang, Tiffany Low, Sin Kei Wong, Emmanuel Guedj, Nikolai V. Ivanov, Walter K. Schlage, Manuel C. Peitsch, Arkadiusz Kuczaj, Patrick Vanscheeuwijck, Julia Hoeng

**Affiliations:** ^1^ Philip Morris International Research and Development, Philip Morris Products S.A. Neuchatel Switzerland; ^2^ Philip Morris International Research Laboratories Pte. Ltd., Science Park II Singapore; ^3^ Biology Consultant Max‐Baermann‐Str. 21 Bergisch Gladbach Germany

**Keywords:** cardiovascular effects, cigarette smoke, nose‐only inhalation, respiratory tract effects, whole‐body inhalation

## Abstract

Cigarette smoking is one major modifiable risk factor in the development and progression of chronic obstructive pulmonary disease and cardiovascular disease. To characterize and compare cigarette smoke (CS)‐induced disease endpoints after exposure in either whole‐body (WB) or nose‐only (NO) exposure systems, we exposed apolipoprotein E‐deficient mice to filtered air (Sham) or to the same total particulate matter (TPM) concentration of mainstream smoke from 3R4F reference cigarettes in NO or WB exposure chambers (EC) for 2 months. At matching TPM concentrations, we observed similar concentrations of carbon monoxide, acetaldehyde, and acrolein, but higher concentrations of nicotine and formaldehyde in NOEC than in WBEC. In both exposure systems, CS exposure led to the expected adaptive changes in nasal epithelia, altered lung function, lung inflammation, and pronounced changes in the nasal epithelial transcriptome and lung proteome. Exposure in the NOEC caused generally more severe histopathological changes in the nasal epithelia and a higher stress response as indicated by body weight decrease and lower blood lymphocyte counts compared with WB exposed mice. Erythropoiesis, and increases in total plasma triglyceride levels and atherosclerotic plaque area were observed only in CS‐exposed mice in the WBEC group but not in the NOEC group. Although the composition of CS in the breathing zone is not completely comparable in the two exposure systems, the CS‐induced respiratory disease endpoints were largely confirmed in both systems, with a higher magnitude of severity after NO exposure. CS‐accelerated atherosclerosis and other pro‐atherosclerotic factors were only significant in WBEC.

## Abstract 2

To characterize and compare cigarette smoke‐induced disease endpoints after exposure in either whole‐body (WB) or nose‐only (NO) exposure chambers (EC), we exposed apolipoprotein E‐deficient mice to filtered air or mainstream smoke from 3R4F reference cigarettes (same target total particulate matter concentration) in NOEC or WBEC for 2 months. The main respiratory disease endpoints and exposure effects were confirmed in both systems, with a greater severity in the NOEC group. Exposure in the WBEC caused generally a more pronounced cardiovascular response.

## 1 INTRODUCTION

Cigarette smoking is one of the major modifiable risk factors in the development and progression of chronic obstructive pulmonary disease (COPD) and cardiovascular disease (CVD) (Centers for Disease Control and Prevention, 2008). In vivo rodent models of cigarette smoke (CS)‐induced COPD and CVD have been shown to be capable of unraveling cellular and molecular disease mechanisms (De Cunto et al., 2020; Kunitomo et al., 2009), and to be suitable for comparative risk assessment of alternative tobacco products (Phillips et al., 2019). Specifically, the apolipoprotein E‐deficient (ApoE^−/−^) mouse model can be used for modeling these CS‐induced diseases as it is susceptible to developing pronounced pulmonary inflammation and increased atherosclerotic plaque progression upon CS exposure (Lo Sasso et al., 2016).

Exposure to CS is typically administered to rodents by using either whole‐body (WB) or nose‐only (NO) exposure systems. During NO exposure, the head and/or nasal regions are primarily exposed, which allows efficient and targeted exposure that limits nonrespiratory exposure routes when compared with WB exposure (Organisation for Economic Co‐operation and Development [OECD], 2018a). Changes or losses of test aerosol constituents attributable to reactions with surfaces of the conducting and exposure tubes can be kept small due to small dead volume and contact surfaces of the whole NOEC setup. During WB exposure, the rodent is surrounded by aerosol, adding to nonrespiratory exposure via grooming of test substance deposited on the fur and/or dermal absorption. In addition, group‐housed animals might huddle together, potentially reducing their inhalation by eventually covering their noses with fur (OECD, 2018b, 2018c; Phalen et al., 1984; Wong, 2007). NO exposure inflicts physical stress upon the animals because of the tube restraint during exposure (Chen & Herbert, 1995; Tuli et al., 1995). Animals in WBECs are probably in less stressful conditions as the rodent is free to move inside the cage. NO exposure limits the number of animals to be exposed, because the procedures involved are time consuming and labor intensive. WBECs allow a large number of subjects to be exposed concomitantly, with practicable labor effort and scale‐up considerations (Mauderly et al., 1989). Therefore, WBECs are commonly used in large and long‐term rodent inhalation studies.

Only few studies to date have compared WB and NO exposure outcomes side by side by using the same concentration of test atmospheres (Mauderly et al., 1989; Oyabu et al., 2016; Shu et al., 2017; Valiulin et al., 2019; Yeh et al., 1990). Mauderly et al. and Shu et al. used CS to expose rats and mice, respectively (Mauderly et al., 1989; Shu et al., 2017). Mauderly et al. reported that after 5 weeks of exposure “parameters thought to be related to chemical carcinogenesis (cell transformation, chromosomal damage, DNA adducts) and chronic lung disease (cell proliferation, inflammation, and respiratory function) were similar” between the two exposure modes, and that “WBEC exposures could achieve greater time‐integrated doses of smoke particulate to the lungs of rats, while reducing stress and toxicity problems” (Mauderly et al., 1989). After 10 weeks of CS exposure plus airway lipopolysaccharides inhalation in C57BL/6 mice, Shu et al. found significantly increased lung inspiratory resistance, functional residual capacity, goblet cell hyperplasia, lung inflammation, and lung angiogenesis for both exposure systems. Changes in right ventricular pressure and intimal thickening of the pulmonary small artery were reportedly a “little more serious” in the NOEC CS exposure group than in the WBEC CS exposure group (Shu et al., 2017).

We have previously performed studies using WBECs to expose ApoE^−/−^ mice to mainstream CS to measure respiratory and cardiovascular disease endpoints (Lietz et al., 2013; Phillips et al., 2016; Phillips et al., 2019; Szostak et al., 2017; Szostak et al., 2020). The use of an NOEC for studying respiratory or cardiovascular endpoints in mice has also been reported previously (Catanzaro et al., 2007; Dekkers et al., 2017; Guo et al., 2006; Lee et al., 2018; Rinaldi et al., 2012; Talukder et al., 2011; Wood et al., 2014).

For this study, our key motivation was to investigate if we can model CS effects on the respiratory and cardiovascular systems in our ApoE^−/−^ disease model equally well using both, WB and NO exposure modes. To this end, we exposed ApoE^−/−^ mice to the same target total particulate matter (TPM) concentration of CS from the 3R4F reference cigarette and, as a control, to filtered air (Sham) in WBECs or NOECs for 2 months. WB and NO exposure effects were characterized with regard to aerosol uptake, adaptive changes in nasal epithelia, changes in lung function, lung proteome, lung and blood inflammatory parameters, plasma cholesterol/triglyceride levels in lipoprotein fractions, atherosclerotic plaque occurrence, and heart transcriptome.

## 2 MATERIALS AND METHODS

### 2.1 General study design

**FIGURE 1:**
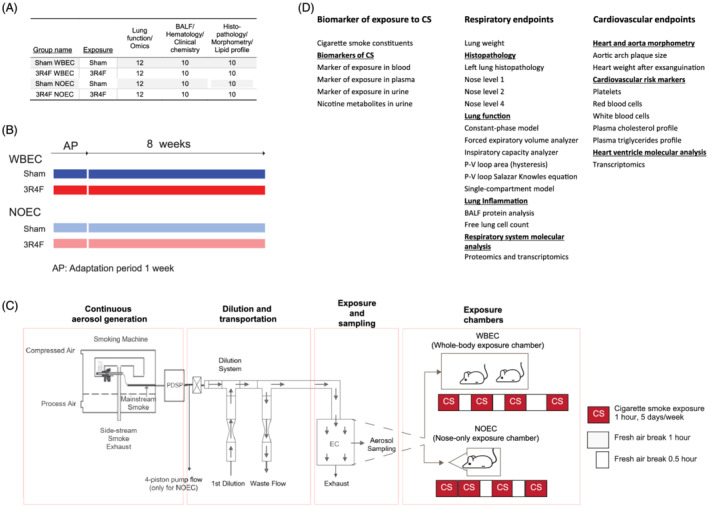
Study and exposure design including fresh air breaks for whole‐body and nose‐only exposure. (A) Group sizes. Mice were randomized into four groups. (B) Study design. (C) Experimental setup for the rodent inhalation study. The output from the programmable dual‐port syringe pump (PDSP) is the PDSP pump flow (see Table 1); two smoking machines were used to generate smoke for the WBEC and one for the NOEC. For figures on the WBEC and NOEC, please refer to Boué et al. (2020) and Lucci et al. (2019), respectively. Different regimens of fresh air breaks were required because of the larger internal volume and, consequently, the longer time, required to clear the smoke from the WBEC. (D) Endpoints examined for evaluating the impact of WBEC and NOEC. BALF, bronchoalveolar lavage fluid; PDSP, programmable dual‐port syringe pump; EC, exposure chamber; NOEC, nose‐only exposure chamber; P–V, pressure–volume; WBEC, whole‐body exposure chamber

This study was conducted to characterize respiratory and cardiovascular endpoints after CS exposure in NOECs and WBECs. Female ApoE^−/−^ mice were randomized into four groups: two Sham groups, exposed to filtered air, and two 3R4F groups, exposed to CS from the 3R4F reference cigarette (550‐μg TPM/L). Half the number of mice in the Sham‐ and CS‐exposed groups were exposed in WBECs and the other half in NOECs (Figure 1A). The exposure phase lasted 9 weeks and included a CS adaptation period, during which exposure in both chamber types was escalated in dose and duration to a maximum of 550‐μg TPM/L for 4 h per day (Figure 1B). The TPM concentration and exposure duration in WBECs were matched to those in NOECs on the basis of the regimen that the mice tolerated, as determined by in‐life findings of acute signs of nicotine toxicity. Fresh air breaks were introduced during the exposure period to maintain carboxyhemoglobin (COHb) concentrations at acceptable levels. More frequent and longer fresh air breaks were required for exposure in the WBEC than in the NOEC because of the greater internal volume and, consequently, the longer duration required to clear the CS from the WBEC. For mice in NOECs, a 30‐min fresh air break was introduced after 2 and 3 h of exposure. For mice in WBECs, a 30‐min fresh air break was introduced after 1 and 2 h of exposure and a 60‐min fresh air break after the third hour of exposure (Figure 1C). At the end of the 2‐month exposure period, mice were euthanized and subsequently dissected to analyze the effect of CS exposure on respiratory and cardiovascular endpoints (Figure 1D).

### 2.2 Test atmosphere generation and characterization

3R4F reference cigarettes were purchased from the University of Kentucky (2003). Mainstream smoke from 3R4F cigarettes was generated on 30‐port rotary smoking machines with 15 ports blocked, active sidestream exhaust (type PMRL‐G, SM2000), and a programmable dual‐port syringe pump (PDSP) in accordance with the Health Canada intense smoking protocol (Health_Canada, 1999), with a puff volume of 55 ml/puff taken in 2 s, puff frequency of one puff every 30 s, and ventilation blocked. Minor deviations from this protocol were necessary for technical reasons; for example, only whole puffs were counted and not rounded to the nearest one‐tenth of a puff (see also Phillips, Veljkovic, et al., 2015).

NOECs and WBECs require significantly different aerosol flow rates because of their design‐related volume differences, and it is rather challenging to perform a comparative study with exactly the same setup for both EC types. Our approach aimed to replicate exactly the same protocols for aerosol generation at the cost of maintaining separate waste flow and dilution factors. The overall goal was to obtain comparable physical and chemical aerosol characteristics for exposure at consequently distinct flow rates. For this reason, two identical smoking machines were used for WBECs and a single one for NOECs, generating an average aerosol flow rate of 3.247 and 1.625 L/min, respectively, each with exactly the same protocol and aerosol characteristics. In NOECs, 1.135 L/min was removed (with a four‐piston pump) to obtain a 0.490 L/min flow rate of undiluted 3R4F mainstream smoke. The 3.247‐ and 0.490‐L/min aerosol flows were diluted with fresh air flow rates of 237.6 and 33.1 L/min, respectively. This resulted in total aerosol flow rates of 240.8 and 33.6 L/min and similar dilution factors of approximately 74‐ and 69‐fold (assuming similar influence on aerosol dynamics) for the WBECs and NOECs, respectively. For WBECs, a flow rate of 98.6 L/min was removed as waste in order to maintain an aerosol flow rate of 142.2 L/min delivered to the exposure chamber (Figure 1C and Table 1). Of note, under the described conditions, the average aerosol residence time is approximately 5.6 min in the WBEC and 0.07 min in the NOEC. With this approach, we matched the delivered TPM concentrations in both exposure chambers at comparatively distinct flow rates.

**TABLE 1:** Aerosol generation parameters

Chamber	Number of smoking machines	PDSP pump flow (L/min)	4‐piston pump flow (L/min)	First dilution (L/min)	Total diluted smoke (L/min)	Waste flow (L/min)	Fold dilution
3R4F WBEC	2	3.247	NA	237.6	240.8	98.6	74
3R4F NOEC	1	1.625	1.135	33.1	33.6	NA	69

*Note*. Total diluted smoke = First dilution + PDSP pump flow − 4‐piston pump flow; Fold dilution = Total diluted smoke / (PDSP pump flow − 4‐piston pump flow).

Abbreviations: 3R4F, reference cigarette; NA, not applicable; NOEC, nose‐only exposure chamber; PDSP, programmable dual‐port syringe pump; WBEC, whole‐body exposure chamber.

The test atmosphere in the aerosol exposure chambers was monitored for flow rate, temperature, relative humidity, particle size distribution, TPM and nicotine concentrations, and concentrations of carbon monoxide (CO), formaldehyde, acetaldehyde, and acrolein, as described previously (Phillips et al., 2016; Phillips, Veljkovic, et al., 2015). In brief, air flow to the chamber was monitored continuously and recorded by using the data acquisition software Wonderware v1415 (INDEFF B.V., Breda, Netherlands). The flow rate for WBECs was adjusted to no less than 120 L/min. The flow through the NOECs was set so that the flow rate per port was at least 0.5 L/min. The air supplied to the smoking machine met the requirements of 60 ± 5% for relative humidity and 22 ± 2°C for temperature during smoking. Conditions inside the smoking machines were not monitored. Test atmospheres were sampled from the exposure chambers at 1 L/min without further dilution of the aerosol sample, and particle size distribution was determined by using a cascade impactor (PIXE I‐1L, PIXE International Corp., Tallahassee, FL, USA). TPM within the exposure chamber was gravimetrically (XS 105 DU, Mettler Toledo, Columbus, OH, USA) analyzed four times per day after trapping on a Cambridge‐type glass fiber filter pad (Pall Corp, Port Washington, NY, USA). Nicotine in smoke was captured four times per day on sulfuric acid‐impregnated 3NT EXtrelut® tubes (Merck Millipore, Burlington, MA, USA). Extraction was performed with 5% v/v trimethylamine in *n*‐butylacetate (Millipore Sigma, St Louis, MI, USA) prior to analysis by capillary gas chromatography (7890A/7890B series, Agilent Technologies, Santa Clara, CA, USA) with a DB‐5 column (Agilent Technologies) by using a flame ionization detector and isoquinoline as the internal standard. CO was continuously monitored by nondispersive infrared photometry (Ultramat 6E, Siemens, Brussels, Belgium) of the gas/vapor phase of the test atmospheres. Aldehyde concentrations were determined by reverse‐phase high‐performance liquid chromatography (HPLC) (1260 series, Agilent Technologies, California, USA) with a Hypersil octadecylsilyl group column (Agilent Technologies) and UV diode array detection of 2,4‐dinitrophenylhydrazine (DNPH) (MilliporeSigma and ITW Reagents, Glenview, IL, USA) derivatives after trapping by bubbling into an impinger containing acid DNPH (3.23 mM)/acetonitrile solution.

### 2.3 CFD modeling

We simulated the aerosol flows inside the exposure chambers by computational fluid dynamics (CFD) analysis and refer the reader to the supporting information section on CFD modeling for further method description.

### 2.4 Animals and inhalation exposure

All procedures involving animals were performed in a facility accredited by the Association for Assessment and Accreditation of Laboratory Animal Care International and licensed by the Agri‐Food & Veterinary Authority of Singapore, with approval from an Institutional Animal Care and Use Committee and in compliance with the National Advisory Committee for Laboratory Animal Research Guidelines on the Care and Use of Animals for Scientific Purposes (NACLAR, 2004). Female ApoE^−/−^ mice (B6.129P2‐ApoE^*tm1/Unc*^ N11) bred under specific‐pathogen‐free conditions were obtained from Taconic Biosciences (Rensselaer, NY, USA). The age and health status of the mice on arrival was verified on the basis of the health check certificate provided by the breeder. Additional details of animal housing, randomization, and acclimatization have been published previously (Boue et al., 2012; Boue et al., 2013; Phillips et al., 2016; Phillips, Veljkovic, et al., 2015). Prior to exposure, the mice were allowed to acclimatize for 1 week. Mice allocated to NO exposure were acclimatized to tube restraint for 5 days, receiving fresh air with incremental restraint time each day. Mice allocated to WB exposure were placed in WBECs for the same length of time. Two 24‐cage WBECs (in‐house design) and two 5 × 12‐port NOECs (CH Technologies, Westwood, NJ, USA) were used (for images of EC refer to Boué et al., 2020 and Lucci et al., 2019). Cage‐enrichment items (e.g., igloo and nesting paper) were provided during the nonexposure period. In WBECs, the nesting paper remained inside the cage during exposure. The cages were changed once a week. The mice were not provided with food during the daily exposure periods. The mice were approximately 9 to 11 weeks of age at the start of the inhalation exposure phase. Exposure in both chamber types began with a concentration escalation and time adaptation, reaching 4 h per day, including weekend exposure during the adaption period. From the second week, exposure was conducted 5 days per week. From study day 17, the mice were exposed to 550‐μg/L TPM for 4 h per day. Fresh air breaks were introduced during the exposure period to maintain COHb concentrations at acceptable levels as described in the general study design and shown in Figure 1C.The maximum time of restraint in the NOEC (including the time for loading and unloading of mice) was 6 h. The general condition and health of the mice following exposure were monitored by daily individual observations.

### 2.5 Biomarkers of exposure

Blood was collected from the facial vein within 15 min postexposure. Blood COHb concentrations in mice were determined at month 1 as described previously (Phillips et al., 2016; Phillips, Veljkovic, et al., 2015). Plasma nicotine and cotinine levels at month 2 were determined by ABF GmbH (Planegg, Germany) as were plasma levels of the oxidative stress marker (lipid peroxidation) malondialdehyde in blood collected immediately after exposure.

Urine was collected during exposure and for approximately 18‐h postexposure in individual metabolic cages at month 2 of the study to obtain 24‐h samples. Urine from mice in the NOEC group was collected during exposure by using adapted restraint tubes before animals were transferred to metabolic cages. In the WBEC group, urine was collected during exposure from mice in single housing in modified cages with a raised grid and after exposure from mice housed in individual mouse metabolic cages.

Analysis of nicotine metabolites (trans‐3′‐hydroxycotinine, norcotinine, cotinine, nicotine‐*N′*‐oxide, and nornicotine) in urine was performed by HPLC after 1,3‐diethyl‐2‐thiobarbituric acid derivatization. The following biomarkers of exposure were assessed by ABF GmbH: 3‐hydroxypropylmercuric acid (HPMA), exposure marker of acrolein; (total) 4‐(methylnitrosamino)‐1‐(3‐pyridyl)‐1‐butanol (NNAL), exposure marker of 4‐(methylnitrosamino)‐1‐(3‐pyridyl)‐1‐butanone (NNK); *S*‐phenylmercapturic acid (SPMA), exposure marker of benzene; 2‐cyanoethyl‐mercapturic acid (CEMA), exposure marker of acrylonitrile; and hydroxybutenyl mercapturic acid (1‐MHBMA) and dihydroxybutyl mercapturic acid (2‐MHBMA), exposure markers of 1,3‐butadiene.

### 2.6 In‐life observations, body weight, and necropsy

The general condition and health of the mice following exposure were monitored throughout the study. This included body weight measurements three times per week until study day 33 and once a week from study day 36. At the end of the 2‐month exposure, the mice were sacrificed before dissection. Necropsy was performed 16–20 h after the last exposure without prior fasting. The mice were anesthetized with 100‐mg/kg pentobarbital (Jurox, Rutherford, NSW, Australia) via intraperitoneal injection. Following blood collection, the mice were exsanguinated via the abdominal aorta. Organ weights of the brain, heart, lungs, and liver were determined from all mice scheduled for dissection (for results, see please refer to https://doi.org/10.26126/intervals.fl34h3.1).

### 2.7 Analysis of lung function

Lung function measurements were performed at the end of month 2, at approximately 18 to 24‐h postexposure. Mice were anesthetized, tracheotomized, and connected to the flexiVent™ rodent ventilator system for measurement of respiratory mechanics (SCIREQ, Montreal, QC, Canada), as previously described (Phillips et al., 2016; Phillips, Veljkovic, et al., 2015). The mice were treated with 0.6 mg/kg rocuronium bromide (MSD, Kenilworth, NJ, USA), a muscle relaxant, before lung mechanics were recorded with the flexiVent equipment and flexiWare 7 software (SCIREQ). The lung volume was recorded as inspiratory capacity obtained during the deep inflation, where the lungs were slowly inflated from positive end‐expiratory pressure (lung pressure at the end of expiration) to 30‐cm H_2_O. Resistance, elastance, and compliance were measured by using the SnapShot‐150, single‐compartment model (SCIREQ), where a single frequency of forced oscillation waveform was applied. Newtonian resistance, tissue damping (tissue resistance), tissue elastance, inertance, and tissue hysteresivity were measured by using the Quick Prime‐3 constant‐phase model (SCIREQ), where multifrequency forced oscillation waveforms were applied. Quasi‐static pressure–volume [P–V] loops were determined from multifrequency forced oscillation waveform consisting of slow stepwise or continuous inflation and deflation cycles. Quasi‐static compliance, quasi‐static elastance, and the area in pressure–volume loops were measured by using the Salazar–Knowles equation. Forced expiratory volume (FEV) at 0.05, 0.10, and 0.20 s and forced vital capacity (FVC) were measured by negative pressure forced expiration. The perturbations were performed at least three times consecutively per animal. Data representation is also shown at https://doi.org/10.26126/intervals.fl34h3.1.

### 2.8 Histoprocessing and histopathological evaluation of the lungs

The lungs were fixed by instillation with an ethanol–glycerol–acetic acid–formaldehyde solution (4% [w/v] formaldehyde; pH 7.4) at a fixed pressure (15‐cm H_2_O) and processed as described previously (Boue et al., 2013). The left lung lobe was longitudinally serially sectioned (4‐μm paraffin sections) into approximately 20 step sections (150‐μm apart) for overall assessment of the entire lung lobe. Representative frontal sections, including the main bronchus, were stained with hematoxylin and eosin (HE), Alcian blue–periodic acid–Schiff reagent (AB‐PAS; for polysaccharides and glycoproteins, including mucus), and resorcin–fuchsin (ResFu; for elastic fibers). Three predefined section levels were used for the nose: posterior to upper incisors (nose level 1); posterior to incisive papilla (nose level 2); and at the first molar teeth (nose level 4); these sections were stained with AB‐PAS and HE. At nose level 1, respiratory epithelial cells including goblet cells (stained using AB‐PAS) were evaluated at the septum; respiratory epithelial cells were evaluated at the lateral wall and turbinates; respiratory and squamous epithelial cells were evaluated at the ventral aspect; respiratory epithelial cells that lined the nasal lumen were evaluated. At nose level 2, respiratory epithelial cells located at the septum, lateral walls, turbinates, and nasal lumen were evaluated; olfactory epithelial cells located at the dorsal aspect/meatus and nasal lumen were evaluated; submucosal gland was evaluated. At nose level 3, olfactory epithelial cells were evaluated at the septum, lateral wall, turbinates, and nasal lumen; olfactory lobe was evaluated at the dorsal aspect, and pharyngeal duct was evaluated at the ventral aspect. Histopathological evaluation of the left lung, nose, and aortic root was performed in a blinded fashion by the study pathologist (Histovia GmbH, Overath, Germany). Incidences of histopathology findings were recorded, and the severity was scored based on a five‐step semiquantitative severity grading: score 0, equal to the morphology of untreated animals; score 1, minimal alteration; score 2, mild, that is, minimal to moderate alteration; score 3, moderate alteration; score 4, marked, that is, moderate to severe alteration; and score 5, severe alteration.

### 2.9 Collection and analysis of BALF

Lavage and free lung cell analysis procedures were described previously (Boue et al., 2013). Cell numbers and viability were determined in native aliquots, whereas the differential counts (macrophages, neutrophils, lymphocytes, and dendritic cells) were evaluated after formaldehyde fixation. The supernatant of the first lavage cycle was used for Luminex®‐based multianalyte profiling (MILLIPLEX® MAP Mouse panels MCVD1MAG‐77K and MCYTOMAG70K, Merck KGaA, Darmstadt, Germany).

### 2.10 Lung processing for proteomics analysis

WB perfusion with cold saline was performed prior to organ removal. Proteins were extracted from one of the right lung lobes as previously described (Lee et al., 2018; Phillips, Veljkovic, et al., 2015). The protein suspensions (50 μg) were processed by using the iTRAQ® 8‐plex labeling procedure in accordance with the manufacturer's instructions (AB Sciex, Framingham, MA, USA). The samples were analyzed in random order by using an Easy nanoLC 1000 instrument (Thermo Fisher Scientific, Waltham, MA, USA) connected online to a Q Exactive™ mass analyzer (Thermo Fisher Scientific). Each sample was injected twice, with two different analytical methods (one fast and another sensitive) on the same column, as previously described (Kelstrup et al., 2014). The outputs of both mass spectrometry runs were combined as merged mass lists and interrogated against the mouse reference proteome set (UniProt, version July 2014, canonical isoforms only) by using Proteome Discoverer v1.4 (Thermo Fisher Scientific). SequestHT implemented in Proteome Discoverer was used as the search tool, and iTRAQ® reporter‐ion intensities were determined from Proteome Discoverer. The Percolator node of Proteome Discoverer was used to estimate peptide‐level false discovery rate (FDR)‐adjusted *p* values (*q* values).

iTRAQ peptide‐level quantification data were exported and further processed in the R statistical environment (R Development Core Team, 2018). The quantification data were filtered for a *q* value < 0.01 and for “unique” quantification results as defined in Proteome Discoverer. Global variance stabilizing normalization was performed with the corresponding Bioconductor package in R (Huber et al., 2002; Hultin‐Rosenberg et al., 2013). Each iTRAQ reporter‐ion set was normalized to its median, and protein expression values were calculated as the median of these normalized peptide‐level quantification values (Herbrich et al., 2013). For detecting differentially abundant proteins, a linear model was fitted for each group comparison, and *p* values were calculated from moderated *t*‐statistics with the empirical Bayes approach (Gentleman et al., 2004). The Benjamini–Hochberg (B–H) FDR method was then used to correct for multiple testing effects. Proteins with adjusted *p* values < 0.05 were considered differentially abundant.

Strengths and limitations of proteomics‐supported toxicology assessment were summarized in previous reviews (Suman et al., 2016; Titz et al., 2014). The main strength of the isobaric‐tagging‐based proteomics approach employed in this study is its quantitative performance and reproducibility (Titz et al., 2014). It is also important to note that broader proteome coverage would be beneficial (Mertins et al., 2018) and that bulk tissue measurements cannot clearly assign cell type specific effects (Slavov, 2020). However, omics analysis allows to uncover impacted pathways in an untargeted manner and allow to elucidate mechanistic insights beyond selective marker analysis.

### 2.11 RNE and heart ventricle processing for transcriptomics analysis

WB perfusion with cold saline was performed prior to organ removal. Respiratory nasal epithelium (RNE) for transcriptomics analysis was isolated from the anterior left side of the nose. The right ventricular chamber of the heart was separated from the left ventricular chamber. The left ventricle was further dissected into two equal‐sized ventral and dorsal parts. The ventral part, further trimmed into smaller sections, was dedicated to transcriptomic analysis. RNA was isolated by using an miRNeasy extraction kit protocol (Qiagen, Hilden, Germany) and further processed by following the GeneChip™ 3′ IVT PLUS protocol (Thermo Fisher Scientific, Waltham, MA, USA). Hybridization was performed on a GeneChip Mouse Genome 430 2.0 array (Thermo Fisher Scientific). Our attempt at isolating good quality RNA from lung tissue failed because of technical reasons.

Raw CEL files were background‐corrected, normalized, and summarized by frozen robust microarray analysis (McCall et al., 2010). Background correction and quantile normalization were performed to generate microarray expression values from all arrays that passed quality control checks, which were performed by using the custom chip description file (CDF) environment Mouse4302_Mm_ENTREZG v16.0 (Dai et al., 2005). Quality control procedures—including analysis of log‐intensities, normalized‐unscaled standard error, relative log expression, median absolute relative log expression value, and pseudoimages as well as raw image plots—were performed with the affyPLM package (Bolstad et al., 2005). Following the quality control procedures, raw *p* values were generated for the group comparisons by using the limma package (Smith et al., 2016) and adjusted using the B–H FDR multiple test correction (Gentleman et al., 2004).

### 2.12 Hematology and blood lipids analysis

Blood was collected from the retro‐orbital venous sinus by using nonheparinized capillary tubes, with the mice under (terminal) pentobarbital anesthesia at the end of the 2‐month exposure. For hematological parameters, blood was collected in ethylenediaminetetraacetic acid (EDTA) tubes. Blood platelet, erythrocyte, hematocrit, and hemoglobin counts/levels were analyzed by using a Sysmex XT‐2000i analyzer (Sysmex Corp., Kobe, Japan) as described previously (Phillips et al., 2016; Phillips, Veljkovic, et al., 2015). Plasma cholesterol (chylomicron, very low‐density lipoprotein [VLDL] cholesterol, low‐density lipoprotein [LDL], and high‐density lipoprotein [HDL]) and triglyceride levels were determined by Immuno‐Biological Laboratories Co., Ltd (Gunma, Japan) by gel permeation size fractionation chromatography followed by enzymatic detection. The analytes were prioritized on the basis of previously reported aerosol exposure‐dependent changes (Coggins et al., 2011; Terpstra et al., 2003; Vanscheeuwijck et al., 2002; Werley et al., 2008). Single analysis and prioritization was necessary because of the low volume of blood available.

### 2.13 Atherosclerotic plaque analysis

The aortic arch of the allocated mice was collected after 2 months of exposure. The aortic arch was flushed with saline, microdissected, cut longitudinally, and then pinned onto a rubber surface. The arch was imaged, stained with Oil Red O, and reimaged to generate both unstained and stained images. The Visiopharm image analysis software (v6.6.1.252; Visiopharm®, Hoersholm, Denmark) was used to determine the areas of the plaques. By using software macros, the perimeter of the aortic arch and the stained regions were outlined, and these borders were confirmed and refined manually. The final relative plaque area, as a percentage, was calculated by dividing the plaque area by the total area of evaluation.

Additionally, a morphometric and a histopathology method were used to determine the atherosclerotic plaque area and composition at the aortic root (for results, see please refer to https://doi.org/10.26126/intervals.fl34h3.1). All evaluations were performed in a blinded manner.

### 2.14 Computational analysis of omics data

By leveraging our “cause‐and‐effect” network models, describing the molecular mechanisms underlying essential biological processes in nondiseased respiratory tissues (Boué et al., 2015; Hoeng et al., 2012), together with network perturbation amplitude (NPA) algorithms, gene expression fold changes were translated into differential values for each network node (Martin et al., 2012; Martin et al., 2014). These were, in turn, summarized into a quantitative NPA measure, and NPA values were aggregated into a biological impact factor (details have been described elsewhere, e.g., Kogel et al., 2014 and Phillips, Veljkovic, et al., 2015).

Gene‐set analysis (GSA) was conducted with the c2.cp gene‐set collection from mSigDB (v5.0) (Liberzon et al., 2011). Two GSA approaches, Camera/Q1 (Wu & Smyth, 2012) and Roast/Q2 (Wu et al., 2010), and over‐representation analysis (Varemo et al., 2013) were applied and jointly evaluated. Q1 tests for the significance of genes in the set versus those not in the set. Q2 tests for a significant difference between the conditions. With this, Q2 is more appropriate in the context of comparative assessment (e.g., to reveal a significant effect of exposure on a given gene set), whereas Q1 can prioritize gene sets that dominate these responses. *P* values were adjusted by Benjamini–Hochberg FDR multiple test correction (Benjamini et al., 2001). FDR‐adjusted *p* values < 0.05 were considered significant.

Pathway analyses of differentially expressed genes were performed by using Qiagen's Ingenuity Pathway Analysis® software (IPA®) with the FDR cutoff set at <0.05 and the fold‐change cutoff at >1.2. The core analysis was performed with the software version from May 2019. The most impacted canonical pathways were scored by using the B–H multiple testing correction *p* value, and entities with –log(B–H) *p* value > 1.3 were selected. The most significant pathways were displayed and colored according to the *Z*‐score (Krämer et al., 2014), which represents the statistical measure of the match between the expected relationship direction and observed gene expression.

### 2.15 Statistical analysis (non‐omics endpoints)

Unless otherwise indicated, data are expressed as mean ± standard error of the mean. The comparative experiment was a two‐factor design comprising: “Smoke exposure” (3R4F vs. Sham) and “Exposure system” (NOEC vs. WBEC). Group comparisons were performed as two sample tests (i.e., in models including only two groups at a time) to more easily manage the potential variance heterogeneity and non‐normality cases through a decision tree. Discrete ordinal scale data were analyzed by the Mann–Whitney–Wilcoxon rank‐sum test, and continuous ratio scale data were analyzed by two‐sample *t*‐tests, assuming nonequal variances (Satterthwaite correction).

For continuous ratio scale data, if, a posteriori, the data showed an obvious departure from normal distribution (assessed by the Shapiro–Wilk test at 5% applied on the Pearson residuals of both groups being compared), a logarithmic data transformation was performed, and the data were analyzed accordingly if this transformation improved the data normality. Otherwise, a nonparametric test was used (Mann–Whitney–Wilcoxon rank‐sum test).

This study was exploratory, and the results were considered as noteworthy finding if, in a specific comparison, the raw *p* value was below the threshold of 5%. All analyses were performed by using SAS 9.2 (SAS Institute, Cary, NC, USA). Our statistically significant key findings are summarized in Figure S1.

### 2.16 Data availability

The transcriptomics data have been deposited in the ArrayExpress public repository (E‐MTAB‐9248). The mass spectrometry proteomics lung data have been deposited in the database of the ProteomeXchange Consortium (Vizcaino et al., 2014) via the PRIDE partner repository with the dataset identifier PXD018185. Other datasets and additional endpoints as well as data visualizations are available on the INTERVALS platform at https://doi.org/10.26126/intervals.fl34h3.1.

## 3 RESULTS

### 3.1 Test atmosphere characterization

**FIGURE 2:**
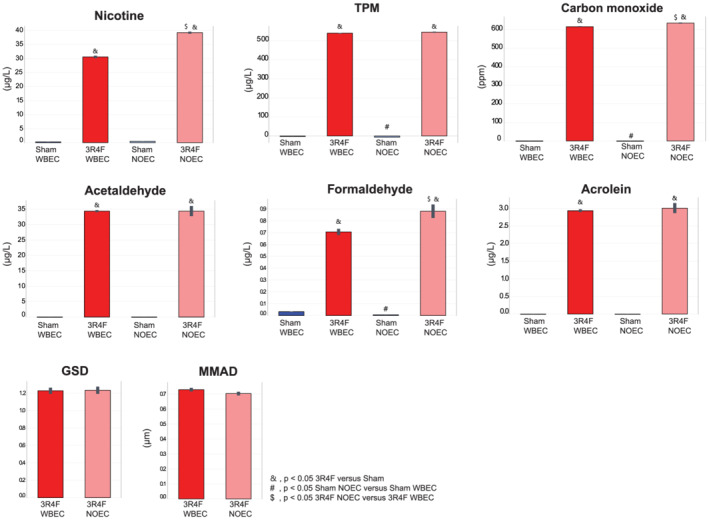
Characteristics of test atmospheres at the breathing zone in the exposure chambers. Values are mean ± standard error of the mean. Symbol “&” denotes statistically significant differences between the test atmosphere (3R4F) and fresh air (Sham) groups (raw *p* < 0.05). “#” denotes statistically significant differences between the Sham groups in the NOEC and WBEC (raw *p* < 0.05). $ denotes statistically significant differences between 3R4F groups in the NOEC and WBEC (raw *p* < 0.05). 3R4F, reference cigarette; GSD, geometric standard deviation (unitless); MMAD, median mass aerodynamic diameter; NOEC, nose‐only exposure chamber; TPM, total particulate matter; WBEC, whole‐body exposure chamber

On the basis of the target TPM concentration of 550 μg/L, CS was reproducibly generated and delivered to the exposure chambers with relative standard deviations of less than 10% for TPM in the test atmospheres (Figure 2). The particle size distribution in the NOEC and WBEC systems had a similar median mass aerodynamic diameter (MMAD) and geometric standard deviation (GSD) that were within the ranges recommended by the Organisation for Economic Co‐operation and Development guidelines (OECD, 2018b, 2018c). Acetaldehyde, acrolein, and CO concentrations were similar in WBECs and NOECs at similar TPM concentrations. Of note, CS in NOECs contained approximately 28% higher nicotine (39.14 ± 0.33 μg/L vs. 30.51 ± 0.43 μg/L) and approximately 24% higher formaldehyde concentrations than the CS in WBECs (0.88 ± 0.06 μg/L vs. 0.71 ± 0.03 μg/L), (Figure 2).

When calculating the yields (i.e., the measured concentration relative to the nominal concentration), the TPM and acrolein yields for NOECs and WBECs were similar, but the nicotine yield in the NOEC was about 13% higher than that in the WBEC (Table S1). The yields for formaldehyde were about 6.5% higher in the NOEC than in the WBEC and in the same range as those for acetaldehyde.

### 3.2 CFD modeling

The aim of applying CFD simulations was to examine the contribution of the exposure chamber geometries, physical aerosol properties, and flow conditions on potentially significant deposition losses inside these systems resulting in nonuniform aerosol. For NOECs, the CFD simulation revealed aerosol separation on the top of the channel delivering the aerosol to the exposure trumpet only for larger particles (>3 μm), which would be beyond any size recommendation for inhalation studies (Lucci et al., 2019). In WBECs, our analysis showed spatial aerosol separation for larger particles (>3 μm) (Figure S2). Furthermore, some aerosol nonuniformity occurred at the bottom of the chamber, with some regions of less concentrated aerosol linked to the flow structures. Of note, animal movement was not included in the modeling. In general, the simulation did not reveal any surprising physical effects (e.g., excessive deposition losses or aerosol separation) related to aerosol delivery that could have an influence on the animal exposure (for further details, refer to the Data S1).

### 3.3 Biomarkers of CS exposure

We monitored CS uptake of the mice by measuring COHb, nicotine, and cotinine concentrations in blood and representative nicotine metabolites and additional exposure biomarkers in urine (Table 2). For both exposure systems, all measured markers were, as expected, significantly higher in concentration in CS‐exposed than in Sham‐exposed mice. The levels of COHb, as a marker of CO concentrations, were approximately 32% (~1.3‐fold) higher in the blood of mice exposed to CS in the NOEC compared with WBEC (Table 2). Nicotine and cotinine levels in plasma were 2.7‐ and 2.4‐fold higher in CS NOEC‐exposed than in WBEC‐exposed mice, respectively. In contrast, total nicotine metabolite levels (excluding nicotine itself) were about sixfold higher in the urine of mice exposed in the WBEC, with a similar fold difference across all individual nicotine metabolites. Part of the nicotine that could be detected in the urine of CS‐exposed mice in WBECs was likely extraneous due to aerosol deposited on cage surfaces and then carried over into the urine samples that were collected from the cage surfaces at the end of exposure. The amount of nicotine detected in the urine of CS‐exposed NOEC mice was much lower than that in urine collected from WBEC mice.

**TABLE 2:** Biomarkers of smoke uptake in blood and urine

			Sham WBEC	3R4F WBEC	Sham NOEC	3R4F NOEC
Aerosol uptake and exposure	Marker of exposure	COHb (%)	2.70 ± 0.06	26.93 ± 1.60^a^	2.57 ± 0.03	35.43 ± 1.38^a^ ^c^
	Marker of exposure in plasma	Cotinine (ng/ml)	0.88 ± 0.00	261.04 ± 43.34^a^	0.88 ± 0.00	636.40 ± 68.40^a^ ^c^
		Nicotine (ng/ml)	2.92 ± 1.03	166.73 ± 31.74^a^	1.25 ± 0.38	457.45 ± 129.65^a^
	Nicotine metabolites in urine (absolute)	Cotinine (nmol)	0.02 ± 0.01	28.91 ± 4.48^a^	0.06 ± 0.03^b^	9.61 ± 1.91^a^ ^c^
		Nicotine (nmol)	0.32 ± 0.15	560.27 ± 265.21^a^	0.47 ± 0.21	27.17 ± 10.26^a^ ^c^
		Nicotine‐1‐*N*‐oxide (nmol)	0.02 ± 0.01	75.89 ± 6.39^a^	0.06 ± 0.02	14.08 ± 2.20^a^ ^c^
		Norcotinine (nmol)	0.01 ± 0.00	19.77 ± 2.62^a^	0.02 ± 0.01^b^	6.18 ± 0.78^a^ ^c^
		Nornicotine (nmol)	0.12 ± 0.01	13.93 ± 1.78^a^	0.16 ± 0.04	4.95 ± 1.00^a^ ^c^
		Total metabolites (nmol)	0.28 ± 0.04	974.92 ± 166.90^a^	0.68 ± 0.19	164.29 ± 16.14^a^ ^c^
		Trans‐3‐hydroxycotinine (nmol)	0.11 ± 0.05	836.41 ± 157.55^a^	0.38 ± 0.19	129.46 ± 12.55^a^ ^c^
	Marker of exposure in urine (absolute)	CEMA (ng)	2.50 ± 0.35	584.05 ± 46.83^a^	2.27 ± 0.33	1,003.35 ± 184.36^a^
		HPMA (ng)	1,722.51 ± 228.53	7,894.53 ± 895.37^a^	1,906.84 ± 308.16	7,268.65 ± 1,173.80^a^
		MHBMA1 (ng)	0.40 ± 0.14	262.98 ± 28.37^a^	6.52 ± 2.72^b^	276.60 ± 30.59^a^
		MHBMA2 (ng)	0.11 ± 0.02	73.00 ± 7.02^a^	0.44 ± 0.21^b^	58.67 ± 5.56^a^
		SPMA (ng)	0.61 ± 0.09	226.78 ± 27.08^a^	0.86 ± 0.09	233.17 ± 33.23^a^
		Total NNAL (pg)	14.63 ± 0.62	266.55 ± 36.15^a^	21.23 ± 1.61^b^	136.29 ± 43.26^a^ ^c^
	Urine creatinine	Creatinine (μmol/L)	3,757.56 ± 411.01	3,736.59 ± 301.16	2,854.21 ± 222.37	3,270.39 ± 437.85
		Total creatinine (μmol)	1,500.39 ± 177.39	2,652.45 ± 274.90^a^	2,112.26 ± 257.33	1,725.79 ± 232.94^c^
		Urine + flush water volume (μl)	511.36 ± 37.74	866.88 ± 121.42^a^	NA	NA
		Urine volume (μl)	411.36 ± 37.74	766.88 ± 121.42^a^	832.50 ± 63.08^**b**^	659.13 ± 60.49

*Note*. Values are mean ± standard error of the mean.

Abbreviations: CEMA, 2‐cyanoethyl‐mercapturic acid; COHb, carboxyhemoglobin; HPMA, 3‐hydroxypropylmercuric acid; NA, not applicable, NNAL, 4‐(methylnitrosamino)‐1‐(3‐pyridyl)‐1‐butanol; MHBMA1, hydroxybutenyl mercapturic acid; MHBMA2, dihydroxybutyl mercapturic acid; NOEC, nose‐only exposure chamber; SPMA, S‐phenylmercapturic acid; WBEC, whole‐body exposure chamber.

^a^ Statistically significant differences between the test atmosphere (3R4F) and fresh air (Sham) groups (raw *p* < 0.05).

^b^ Statistically significant differences between the Sham groups in the NOEC and WBEC (raw *p* < 0.05).

^c^ Statistically significant differences between 3R4F groups in the NOEC and WBEC (raw *p* < 0.05).

The levels of HPMA (metabolite of acrolein), SPMA (metabolite of benzene), CEMA (metabolite of acrylonitrile), and 1‐MHBMA and 2‐MHBMA (metabolites of 1,3‐butadiene) were similar or slightly higher in the urine of NOEC‐exposed mice than in urine from mice exposed in WBECs. Only the total NNAL (metabolite of NNK) level was lower in the urine of NOEC‐exposed mice compared with that of mice exposed in WBECs (Table 2).

### 3.4 In‐life observations after CS exposure

Postexposure examination revealed tremors, mild and transient in nature, in mice that were exposed to CS in NOECs but not in Sham‐exposed mice in NOECs or in any of the WBEC groups, which is consistent with higher plasma nicotine in the CS NOEC group. A low incidence of breathing difficulty and Straub tail were also observed in mice that were exposed to CS in NOECs (data not shown).

**FIGURE 3:**
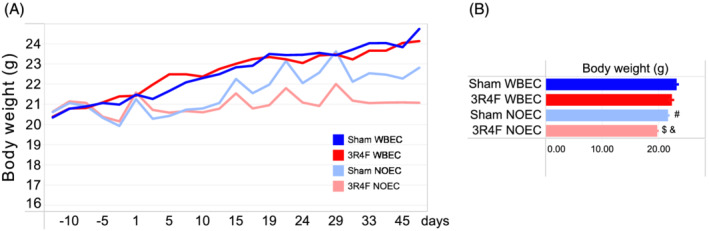
Body weight. (A) Mean body weight over time. (B) Mean body weight at the end of the exposure period. 3R4F, reference cigarette; NOEC, nose‐only exposure chamber; WBEC, whole‐body exposure chamber

Both Sham‐ and CS‐exposed mice in the WBEC gained weight during the 2‐month study (Figure 3A). Sham‐ and CS‐exposed mice in the NOEC lost body weight during the first 2 weeks of exposure. Although Sham‐exposed mice gained body weight over time, no such gain was apparent in CS‐exposed mice in the NOEC. However, mice exposed in NOECs showed a slight weight gain after the weekend exposure breaks (indicated as peaks in the body weight graph), which again decreased during the exposure days. At the end of exposure, the Sham‐exposed mice in the NOEC had a 6% lower terminal body weight than those in the WBEC. CS exposure led to a further decrease in body weight of 9.2% in NOEC‐exposed mice compared with Sham‐exposed mice; whereas only a 3.4% reduction was observed in CS‐exposed mice relative to Sham‐exposed mice in the WBEC group (Figure 3B). Overall, body weight was significantly higher in WBEC‐exposed mice than in NOEC‐exposed mice.

### 3.5 Effect of CS exposure on structural and molecular changes in the upper respiratory tract

**FIGURE 4:**
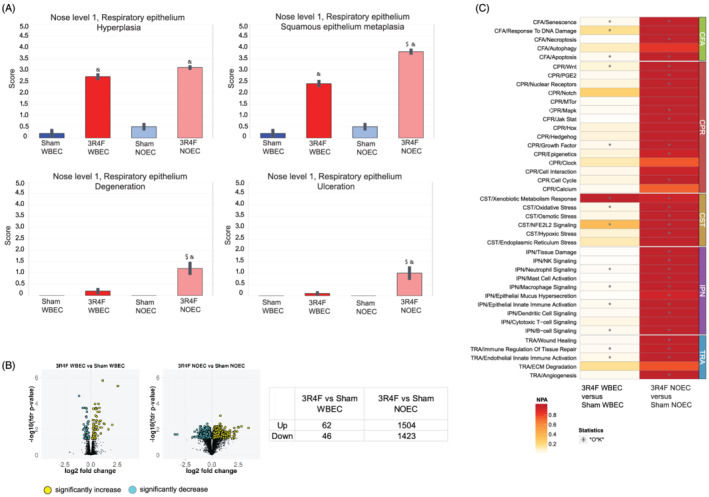
CS exposure in WBEC and NOEC induces histopathological and molecular changes in the upper respiratory tract with a higher amplitude in NOEC. (A) Histopathological findings in nose level 1. Mean severity scores ± standard error of the mean are shown. Symbol “&” denotes statistically significant differences between the test atmosphere (3R4F) and fresh air (Sham) groups (raw *p* < 0.05). “#” denotes statistically significant differences between the Sham groups in the NOEC and WBEC (raw *p* < 0.05). “$” denotes statistically significant differences between 3R4F groups in the NOEC and WBEC (raw *p* < 0.05). Histopathological findings of nose level 2 and 4 are shown in Table S2. For incidences of histopathological findings refer to Table S3. (B) Gene expression analysis of respiratory nasal epithelium. Numbers of differentially expressed genes are shown in volcano plots and tabulated. (C) Heatmap of Network Perturbation Amplitude (NPA) Scores. A network is considered perturbed if, in addition to the significance of the NPA score with respect to the experimental variation, the two companion statistics (O and K) derived to inform on the specificity of the NPA score with respect to the biology described in the network are significant. The NPA scores are normalized per row, that is, per biological process. Asterisk “*” denotes O and K statistics *p* < 0.05. 3R4F, reference cigarette; CFA, cell fate; CPR, cell proliferation; CST, cell stress; ECM, extracellular matrix degradation; IPN, inflammatory process networks; NOEC, nose‐only exposure chamber; TRA, tissue repair and angiogenesis; WBEC, whole‐body exposure chamber

Nose histopathological findings in WB CS‐exposed mice encompassed mild to moderate hyperplasia and squamous epithelial metaplasia of the respiratory epithelium at nose level 1. These findings were not observed at nose level 2. Respiratory epithelial hyperplasia and squamous epithelial metaplasia of respiratory epithelium were moderate to marked at nose level 1 and minimal to moderate at nose level 2 in NO CS‐exposed mice. Degeneration and ulceration of the respiratory epithelium, as well as atrophy of the olfactory epithelium and loss of nerve bundles at the lamina propria of the olfactory epithelium were of low incidence but not statistically significantly different in the CS‐exposed WBEC group compared with Sham. In contrast, mice exposed to CS in the NOEC exhibited more severe findings and in deeper levels of the nose than mice exposed in the WBEC (Figure 4A, Tables S2 and S3). Degeneration and ulceration of the respiratory epithelium as well as loss of nerve bundles at the lamina propria of the olfactory epithelium was observed at nose level 2 in NO CS‐exposed mice, whereas mild to moderate atrophy of the olfactory epithelium was observed at nose level 4 of the NO CS‐exposed mice.

Gene expression analysis revealed 108 differentially expressed genes (FDR‐adjusted *p* value < 0.05) in the RNE transcriptome of CS‐exposed mice in the WBEC compared with the corresponding Sham exposure group; in contrast, in the NOEC, 2,927 genes were differentially expressed (FDR‐adjusted *p* value < 0.05) in response to CS exposure compared with Sham exposure (Figure 4B). The threshold‐free approach of calculating the network perturbation of biological processes that are known to be affected by CS revealed that NO exposure had a bigger impact than WB exposure on all tested biological signaling processes. Only the xenobiotic metabolism response in RNE was perturbed at the same level in the CS‐exposed WBEC group as in the NOEC group (Figure 4C).

### 3.6 Effect of CS exposure on lung function, inflammation, and molecular changes

**FIGURE 5:**
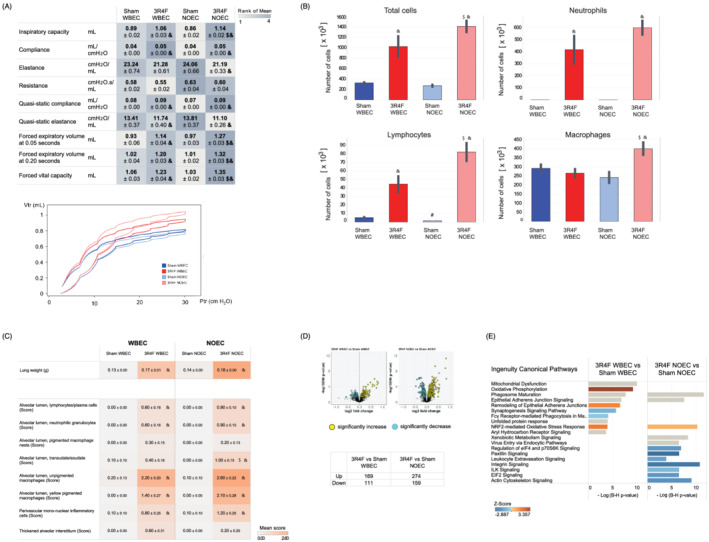
CS exposure in WBEC and NOEC impacts lung function, induces lung inflammation, and dysregulates the proteome. (A) Lung function parameters. Lung volume analysis by inspiratory capacity measurement, snapshot perturbations (single‐compartment model): lung resistance, elastance, and compliance, forced vital capacity, forced expiratory volume at 0.05 and 0.20 s, and pressure–volume loops and parameters. (B) Lung inflammation determined by free lung cell counts in bronchoalveolar lavage fluid (BALF). (C) Absolute lung weight and histopathological evaluation of the left lung. The impact was evaluated by scoring the severity of the findings. For incidences on histopathological findings refer to Table S3. Higher scores are marked with darker colors over all findings. All values are means ± standard error of the mean. Symbol “&” denotes statistically significant differences between the test atmosphere (3R4F) and fresh air (Sham) groups (raw p < 0.05). “#” denotes statistically significant differences between the Sham groups in the NOEC and WBEC (raw p < 0.05). “$”denotes statistically significant differences between 3R4F groups in the NOEC and WBEC (raw p < 0.05). (D) Proteomics analysis of the lungs. Differentially abundant proteins are shown in volcano plots. The numbers of upregulated and downregulated proteins are shown in tabular format. (E) Affected canonical pathways, as determined by Ingenuity Pathway Analysis (IPA). The top 10 pathways are shown. Pathways were ranked for significance according to −log((B–H)‐adjusted *p* value). Coloration corresponds to the *Z*‐score. (QIAGEN Inc., ttps://www.qiagenbioinformatics.com/products/ingenuity‐pathway‐analysis, version May 2019). 3R4F, reference cigarette; NOEC, nose‐only exposure chamber; WBEC, whole‐body exposure chamber

The lung volume, obtained by measuring inspiratory capacity where the lungs are slowly inflated from positive end‐expiratory pressure using the deep inflation maneuver, was significantly higher in both CS exposure groups than in the Sham‐exposed groups, and slightly higher in NOEC CS‐exposed mice than in WBEC CS‐exposed mice (Figure 5A). The total respiratory system resistance (both airway and tissue resistance), elastance (elastic rigidity or stiffness of the lungs; reciprocal of compliance), and compliance from single‐compartment “snapshot perturbation” measurements to represent the sum of airway and alveolar space responses showed that in comparison with Sham exposure, CS exposure elicited a significantly higher compliance and slightly lower elastance and resistance in the NOEC and WBEC groups, with CS exposure being significant in the NOEC group only for elastance. The pressure–volume loops that capture the quasi‐static mechanical properties of the respiratory system showed an upward and leftward shift for both the inflation and deflation phases of the maneuver in response to CS exposure in both WBEC and NOEC mice (Figure 5A).

Inflammation in the lung was observed in mice exposed to CS in WBECs and NOECs: the number of total free lung cells in bronchoalveolar lavage fluid (BALF) was significantly higher in CS‐exposed WBEC and NOEC groups compared with the respective Sham groups and higher in the CS‐exposed NOEC group than in the WBEC group. This higher cell count in the NOEC CS‐exposed group compared with WBEC CS‐exposed group was found for all cell types analyzed: there were significant changes in the numbers of total free lung cells, total lymphocyte, and macrophage counts, as well as trends towards changes in the numbers of neutrophils (Figure 5B). The majority of upregulated inflammatory mediators in the BALF from CS‐exposed mice were common to both WBEC and NOEC exposure (Table S4). Effects of CS exposure on lung weight in comparison with Sham exposure were found for absolute lung weight (and also relative to body or brain weight) in both NOEC‐ and WBEC‐exposed mice (Figure 5C).

Additional histopathological examination of the left lung revealed mild to moderate unpigmented and yellow‐pigmented macrophages in the alveolar lumen of CS‐exposed mice, with a tendency towards higher severity for mice exposed to CS in the NOEC (Figure 5C and Table S3). Perivascular mononuclear cells were reported as minimal to mild and neutrophilic granulocytes and lymphocytes as minimal in both WBEC and NOEC CS‐exposed mice. Only the transudate/exudate in the alveolar lumen had a significantly higher severity score in the CS‐exposed NOEC group compared with the CS‐exposed WBEC group (Figure 5C).

The proteome of the lung tissue was analyzed to gain insights into the molecular mechanisms impacted by CS exposure in the lung tissue. The analysis revealed 280 differentially abundant proteins (FDR‐adjusted *p* value < 0.05) following CS exposure in the WBEC and 433 (FDR‐adjusted *p* value < 0.05) following CS exposure in the NOEC in comparison with the respective Sham controls (Figure 5D). In the WBEC group, the differentially expressed proteins were associated with oxidative phosphorylation/mitochondrial dysfunction, phagosome maturation, epithelial adherens junction signaling, aryl hydrocarbon receptor signaling, and NRF2‐mediated oxidative stress. Similarly, CS exposure in the NOEC affected proteins associated with phagosome maturation, oxidative stress response, xenobiotic metabolism, and epithelial adherens junction signaling pathways (Figure 5E).

### 3.7 Effect of CS exposure on blood lipid profile and lipid peroxidation

**FIGURE 6:**
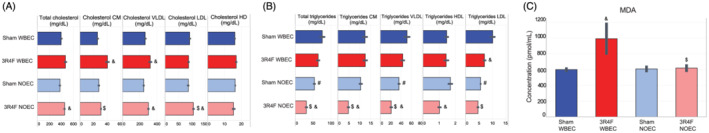
CS exposure significantly impacts plasma triglyceride levels in NO‐exposed mice and plasma oxidative stress marker in WB‐exposed mice. (A) Total and lipoprotein cholesterol levels. (B) Total and lipoprotein triglyceride levels. (C) Malondialdehyde level. All values are mean ± standard error of the mean. Symbol denotes “&” statistically significant differences between the test atmosphere (3R4F) and fresh air (Sham) groups (raw *p* < 0.05). “#” denotes statistically significant differences between the Sham groups in the NOEC and WBEC (raw *p* < 0.05). “$” denotes statistically significant differences between 3R4F groups in the NOEC and WBEC (raw *p* < 0.05). 3R4F, reference cigarette; CM, chylomicron; HDL, high‐density lipoprotein; LDL, low‐density lipoprotein; MDA, Malondialdehyde; NOEC, nose‐only exposure chamber; VLDL, very‐low‐density lipoprotein; WBEC, whole‐body exposure chamber

Analysis of plasma lipids showed that chylomicron and VLDL cholesterol levels were significantly higher after CS exposure in the WBEC group, and VLDL and LDL cholesterol levels were significantly higher after CS exposure in the NOEC group compared with the respective Sham‐exposed groups. Chylomicron cholesterol level was lower, whereas LDL cholesterol was higher after CS exposure in the NOEC group compared with CS‐exposed WBEC group (Figure 6A).

Only LDL triglyceride levels were significantly lower following CS exposure in the WBEC compared with Sham‐exposed mice. Chylomicron, VLDL, and HDL triglyceride levels were significantly lower in CS‐exposed mice in the NOEC. Total, chylomicron, LDL, and VLDL cholesterol levels were significantly lower in CS‐exposed NOEC compared with CS‐exposed WBEC group. Interestingly, total triglyceride levels were lower in the Sham NOEC group than in the Sham WBEC group. This finding is largely attributable to the lower levels of LDL and VLDL triglycerides in Sham mice exposed in the NOEC (Figure 6B).

A significant increase in the concentration of plasma malondialdehyde, a product of lipid peroxidation, was observed in CS‐exposed WBEC mice compared with Sham or CS‐exposed NOEC mice. No significant change relative to the Sham levels was observed in the CS‐exposed NOEC group (Figure 6C).

### 3.8 Effect of CS exposure on red and white blood cells

**FIGURE 7:**
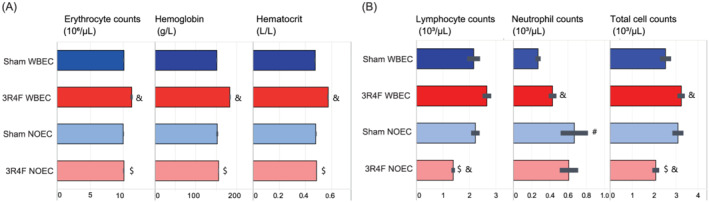
CS exposure significantly impacts red blood cell numbers in WB‐exposed mice and white blood cells in NO‐exposed mice. Hematological findings are shown. (A) Red blood cells. (B) White blood cells. Values are mean ± standard error of the mean. Symbol “&” denotesstatistically significant differences between the test atmosphere (3R4F) and fresh air (Sham) groups (raw *p* < 0.05). “#” denotes statistically significant differences between the Sham groups in the NOEC and WBEC (raw *p* < 0.05). “$” denotes statistically significant differences between 3R4F groups in the NOEC and WBEC (raw *p* < 0.05). 3R4F, reference cigarette; NOEC, nose‐only exposure chamber; WBEC, whole‐body exposure chamber

Red blood cell analysis revealed CS exposure‐related effects that were more pronounced in WBEC‐exposed mice than in those exposed in a NOEC: Erythrocyte number, hemoglobin concentration, and hematocrit levels were significantly higher in these mice than in Sham‐exposed mice (Figure 7A). White blood cell counts were higher in the CS‐exposed WBEC group than in the Sham WBEC group, mostly owing to higher neutrophil counts (Figure 7B). In contrast, white blood cell counts were lower in the CS‐exposed NOEC group than in the Sham NOEC group, mostly owing to lower lymphocyte counts.

### 3.9 Effect of CS exposure on atherosclerotic plaques and molecular changes in the heart

**FIGURE 8:**
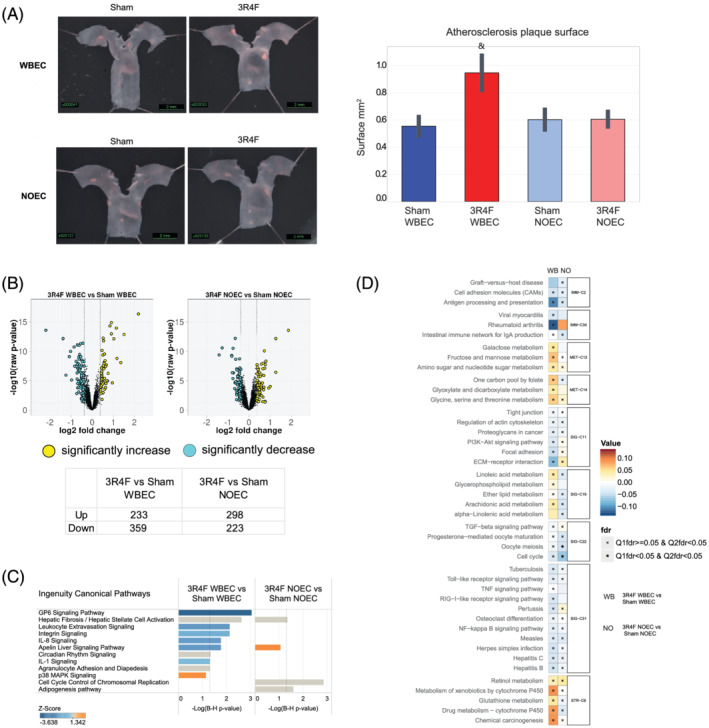
CS exposure in WBEC accelerates significantly atherosclerotic plaques and effects distinct molecular mechanisms in WBEC and NOEC. (A) Representative images of atherosclerotic lesions in the aortic arch, with measurements of the atherosclerotic plaque surface area acquired by planimetry at 2 months. Values are mean ± standard error of the mean. Symbol “&” denotes statistically significant differences between the test atmosphere (3R4F) and fresh air (Sham) groups (raw *p* < 0.05). (B) Volcano plot representing the changes in gene expression in the heart ventricle after exposure to CS. Yellow dots indicate significantly upregulated, and cyan dots indicates significantly downregulated genes; FDR‐adjusted *p* values *p* < 0.05. (C) Affected canonical pathways, as determined by Ingenuity Pathway Analysis (IPA). Canonical pathways above the threshold of −log((B–H)‐adjusted *p* value) < 1.3 are shown and ranked for the top 10 pathways. Coloration corresponds to the *Z*‐score (QIAGEN Inc., https://www.qiagenbioinformatics.com/products/ingenuity‐pathway‐analysis). (D) Gene‐set analysis of the c2.cp collection of mSigDB. 3R4F, reference cigarette; NOEC, nose‐only exposure chamber; WBEC, whole‐body exposure chamber

Two‐dimensional image analysis of the aortic plaque area at the dissected aortic arch revealed a slightly higher plaque area in mice exposed to CS in the WBEC compared with the respective Sham group (0.94 mm^2^ vs. 0.55 mm^2^; *p* < 0.05). No difference was found in aortic plaque area between CS‐ and Sham‐exposed mice in the NOEC group (both 0.60 mm^2^) (Figure 8A).

Gene expression analysis of the left heart ventricle showed that CS exposure caused significant dysregulation of 592 genes (FDR‐adjusted *p* value < 0.05) in the WBEC group and 521 genes (FDR‐adjusted *p* value < 0.05) in the NOEC group (Figure 8B). In mice exposed to CS in the WBEC, these gene expression changes could be attributed to the downregulation of the GP6 (glycoprotein VI) signaling pathway (−log(B–H) *p* value > 1.3), among others, due to downregulation of collagens (COL1A‐ and COL4‐ and COL6‐ types) and laminin subunits (GP6 can serve as a signaling receptor for both proteins) (Figure 8C), and to the downregulation of leukocyte extravasation, integrin, IL‐8 and IL‐1 signaling (−log(B–H) *p* value > 1.3). In mice exposed to CS in the NOEC, these gene expression changes could be attributed to fibrotic events due to the upregulation of collagen 5 types and tissue inhibitors of metalloproteinase 1. The inflammatory pathways that were affected in the CS‐exposed WBEC group were not significantly affected in the CS‐exposed NOEC group.

The GSA associated the differentially expressed genes in the left heart ventricle of mice exposed to CS in the WBEC with several upregulated metabolism pathways as galactose, fructose or linoleic acid, as well as with xenobiotics metabolism, with downregulated “ECM‐receptor interaction” and “Focal adhesion”, and with downregulated inflammatory pathways. In the NOEC‐ group, the differentially expressed genes were associated with downregulated “Cell cycle” (Figure 8D). Interestingly, the directionality of pathway activation in response to CS seemed to be divergent in WBEC and NOEC groups. In fact, “Metabolism of xenobiotics” and “Drug metabolism cytochrome P450” were associated with an activated response to CS exposure in WBEC, but associated with a downregulated response to CS exposure in NOEC.

## 4 DISCUSSION

Our key motivation was to investigate if we can model CS effects on the respiratory and cardiovascular systems in our ApoE^−/−^ disease model equally well using both, WB and NO exposure modes.

The analysis of the test atmospheres in each CS EC confirmed comparable delivery of TPM, CO, acetaldehyde, and acrolein concentrations in WBEC and NOEC. However, nicotine and formaldehyde concentrations and the yields were higher in the NOEC than in WBEC. Changes in the thermodynamic state of the CS aerosol mixture (solid carbonaceous and liquid particles surrounded by gas/vapor equilibrating accordingly to temperature, pressure and local flow velocities) affect its constituent concentrations. The partitioning and equilibration between constituents differ due to distinct aerosol flow rates within the two systems, consequently leading to constituent‐specific differences in measured concentrations. In particular, as the two exposure systems require distinct flow rates, the resulting aerosol average residence times are different and lead to distinct phase‐equilibriums that are dependent on concentration and thermodynamic state. Additionally, the higher nicotine and formaldehyde concentrations, as well as yields, in the NOEC compared with those in the WBEC are likely to be related mainly to filtration losses along the aerosol delivery line (caused by aerosol aging and dilution) and inside both chambers (caused by the available surfaces of materials with distinct sorption properties). The larger surface area in WBECs requires more material to be deposited before equilibrium can be reached. The material components in ECs such as the presence of wood shavings (i.e., from beddings), nesting paper, animal excretions and fur, water bottles, and plastic cages in WBECs have different chemical affinity which may result in a difference in nicotine and aldehyde deposition. NOECs are constructed mainly of stainless steel components, which are expected to be more inert than the materials in WBECs. Also, differences in the spatial homogeneity of aerosol particle number density may affect the measured aerosol constituent concentration. The typical coefficient of variation of aerosol particle number density sampled from different positions can be expected to be <5% for NOECs (Pauluhn, 2003) and <15% (Yeh et al., 1986) for WBECs. A lower coefficient of variation indicates that there is less spatial variability in the aerosol concentration. WBECs, with their greater internal volumes could be less spatially homogeneous than NOECs. As such, sampling from a single position in the WBEC (in this case, the middle section of the WBEC) could induce a bias/artifact in the results.

CFD modeling of aerosol deposition on the basis of physical parameters and geometry did not show significant deposition effects or differences between the NOEC and WBEC in the percentage of deposited aerosols particulates in the (human) inhalable range of particle sizes (i.e., <3 μm) (see Data S1: Computational Fluid Dynamics Modeling). This indicates that future studies on the differences in yield for different aerosol constituents could benefit from focusing on the differences in the physicochemical properties of aerosol constituents (e.g., gas–liquid partitioning and sorption properties).

In sum, the composition of CS in the breathing zone between the two exposure modes is not completely comparable. Additionally, the functional differences of the two systems allow for instantaneous delivery and exchange of aerosol at the nose port in case of NOEC but imply potential for rebreathing of the atmosphere by the animals in the WBEC system.

Biomarkers were measured to understand better the aerosol uptake by the mice. COHb levels were higher in NO CS‐exposed mice than in WB CS‐exposed mice. Because the CO concentrations in the WBEC and NOEC CS atmospheres were similar, the differences in COHb levels in the CS‐exposed mice might be attributable to the longer fresh air breaks given to WBEC‐exposed mice (60 min rather than 30 min).

Plasma nicotine and cotinine levels were higher in the NOEC group than in the WBEC group. The approximately 28% higher nicotine concentration in the aerosol of the NOEC could, in part, explain the higher plasma nicotine and cotinine concentrations in the CS‐exposed mice. The shorter fresh air breaks between exposures in the NOEC would allow higher plasma nicotine and cotinine buildup compared with exposure in the WBEC. Furthermore, because of its small internal volume (inner plenum), the NOEC reaches aerosol saturation more rapidly, which might result in higher net nicotine exposure than in the WBEC. The directed aerosol delivery to the nose of NO‐exposed mice (compared with the potential huddling of group‐housed animals in WBECs) could also result in higher inhalative nicotine uptake in NOECs than in WBECs. Possibly, also a difference in the breathing pattern (e.g., frequency or respiratory minute volume) of mice exposed in WBEC compared with NOEC could explain differences in aerosol uptake. Those measurements are recommended to be included in such future studies. Of note, the plasma nicotine and cotinine levels in the CS‐exposed NOEC group were higher in this study compared with a previous NO inhalation study using C57BL/6 mice with the same aerosol nicotine concentration (about 40 μg/L) (Lee et al., 2018), suggesting mouse strain‐related differences. The plasma nicotine and cotinine levels from mice exposed to CS in WBEC were comparable with previous ApoE^−/−^ studies (Phillips et al., 2016; Phillips et al., 2019).

In contrast to the plasma findings, urinary nicotine metabolite levels were higher in the CS‐exposed WBEC group than in the CS‐exposed NOEC group. The two groups did not differ significantly in total creatinine in 24‐h urine normalized to body weight or urine volume, which suggested similar urine output and/or urine recovery. Transdermal uptake and uptake via grooming and licking from cage surfaces are potential routes to account for the higher uptake of particulate aerosol components in the WB‐exposed animals than in NO‐exposed animals (Tyl et al., 1995; Wolff et al., 1982). In these terms, the total nicotine uptake is reflected in the 24‐h urine, in which the nicotine that was not in the organism at the time point of blood collection, is added via noninhalation routes that occurred after the daily exposure.

In sum, the uptake of CS is not completely comparable between the two exposure modes.

In the nose, a clear difference in the severity of effects was observed between WBEC and NOEC CS exposure. Epithelial degeneration and ulceration were present in CS‐exposed NOEC mice after 2 months of exposure, which is indicative of persistent local toxicity. Epithelial degeneration and ulceration are known effects of short‐term WB CS exposure (Stinn et al., 2010; Stinn et al., 2013) and will normally be replaced by epithelial adaptation within the first week of the exposure period (Monticello et al., 1990). Epithelial degeneration and ulceration cause significant stress to the animals and might negatively impact the analysis of typical adaptive changes that follow. Therefore, extensive epithelial degeneration and ulceration should be avoided by either prolonging the gradual increase in concentration in the adaptation phase in order to generate adaptive changes instead of destroying the epithelium right away or by reducing the exposure concentration or exposure duration per day in long‐term inhalation studies. Additionally, mice exposed to CS in the NOEC exhibited changes that were more severe and extended to deeper levels of the nose than mice exposed to CS in the WBEC. Transcriptome analysis of the nose also indicated that CS exposure in NOECs exerted a higher impact on the epithelium. Importantly, the 2‐month NOEC transcriptome response was similar to the WBEC transcriptome response analyzed at later time points in a study on ApoE^−/−^ mice exposed to CS for 3, 4, and 6 months (Phillips et al., 2019) (Figure S3A). The higher toxicity can possibly be explained by the higher local exposure that occurred in NOECs than in WBECs as outlined above: due to smaller chamber volume, the saturation and equilibrium aerosol concentration was reached more rapidly in NOECs than in WBECs. On the other hand, WB exposure might decrease the inhaled dose through deposition losses (deposition of CS constituents on the fur and the larger contact surfaces of the WBEC, filtering of the aerosol through the fur) (Pauluhn & Mohr, 2000; Phelps et al., 1984; Wong, 2007). Overall, the higher local exposure effects seen in the evaluation of histological and transcriptomic endpoints in the nose of NO‐exposed mice are consistent with the higher COHb and plasma nicotine and cotinine levels, and might also be linked to certain constituents of the CS, such as formaldehyde, that were present at higher concentrations in the NOEC test atmosphere compared to the WBEC.

Assessment of the lungs showed typical inflammatory changes and higher weight following CS exposure, independent of the exposure system and consistent with previously published data (Phillips et al., 2016; Phillips, Veljkovic, et al., 2015; Rangasamy et al., 2009; Tsuji et al., 2015). Inflammation is a key hallmark that drives the pathophysiological changes observed in COPD (De Cunto et al., 2020; Liang & He, 2019; Sharafkhaneh et al., 2008). Infiltrating immune cells, in particular alveolar macrophages and neutrophils, secrete a variety of inflammatory mediators, including cytokines, chemokines, and proteases, which contribute to tissue damage processes as seen in lung emphysema onset. Additionally, results of pressure–volume loops and the values for compliance, elastance, and resistance indicated the beginning of emphysematous changes following CS exposure in WBEC and NOEC. Whereas higher numbers of inflammatory cells in the lung following CS exposure in the NOEC group than the WBEC group were observed, lung histopathological analysis showed only a statistically significantly higher severity score of the transudate/exudate in the alveolar lumen in CS‐exposed mice in NOEC than in those in the WBEC. Even though the incidence of inflammatory cells present in the alveolar lumen was slightly higher in the NO CS‐exposed group, the severity score difference did not reach statistical significance between the WBEC and NOEC groups. The semiquantitative nature of histopathology evaluation could be less sensitive to detect small differences in inflammatory cell counts in the lungs because only one cross section of the left lung was evaluated as opposed to the whole lungs' free lung cells in the BALF analysis.

The lung proteome findings showed that CS exposure in the NOEC elicited a slightly stronger response than that in the WBEC. In general, these proteomics findings were comparable with those of previous studies that reported an upregulation in oxidative stress response and xenobiotic metabolism following WBEC exposure (Phillips et al., 2019). Pulmonary oxidative stress was previously shown to be associated with CS‐induced emphysematous changes (Rangasamy et al., 2004; Rangasamy et al., ).^2009^  As noted with the RNE transcriptome, the lung proteome response observed in this 2‐month NOEC exposure was similar to the WBEC lung proteome response at later time points in a study on ApoE^−/−^ mice exposed to CS for 3, 4, and 6 months (Phillips et al., 2019) (Figure S3B). Further investigations are needed to confirm if the respiratory effects would be equal in WB and NO exposure if the inhalation period in the WBEC were longer. However, significant respiratory effects are noted in both exposure systems in this 2‐month exposure study.

The analysis of cardiovascular endpoints highlighted that CS exposure in NOEC had a significantly lower biological effect on the cardiovascular system than the exposure in WBEC. Evaluation of the aortic arch revealed increased atherosclerotic plaque area in the CS‐exposed WBEC group relative to the Sham group. In contrast, no increase in atherosclerotic plaque area was observed in the CS‐exposed NOEC group, whereas CS exposure in the NOEC was previously reported to increase the area of lipid‐rich aortic lesions after 8 weeks of exposure (Catanzaro et al., 2007). Increase in atherosclerotic plaque size after CS exposure in WBECs has previously been reported in ApoE^−/−^ mice (Gairola et al., 2001; Lietz et al., 2013; Phillips et al., 2016). Although we saw a small but significant increase in atherosclerotic plaque area in the aortic arch from 2 months onwards in the 2016 Phillips et al. study, this increase was significant only from after 3 months of CS exposure in the 2019 Phillips et al. study (Phillips et al., 2016; Phillips et al., 2019). Thus, the 2‐month exposure period delineates likely the beginning of significant CS effects on increasing plaque area.

In addition to the absence of an increased atherosclerotic plaque area in the CS‐exposed NOEC group, we observed also no oxidative stress response in the CS exposed NOEC mice. Malondialdehyde concentration in plasma was not affected by CS exposure in the NOEC group. Our transcriptomics analysis of the heart ventricle demonstrated the activation of xenobiotic metabolism pathways in the heart in response to CS in WBEC, suggesting the activation of CYP genes, which is in accordance with known CS exposure‐induced mechanisms. For example, polycyclic aromatic hydrocarbons identified in CS are oxidized by cytochrome P450 enzymes (e.g., Cyp1A1) and the resultant metabolites exert pro‐oxidant effects on the cardiorespiratory system (Briede et al., 2004; Luckert et al., 2013; Ranjit et al., 2016). However, such a metabolism of xenobiotics was not found in the CS‐exposed NOEC group. In our previous ApoE^−/−^ study in WBEC, we observed similar activation of oxidative stress mechanisms after 6 months (Szostak et al., 2017) or after 4 months of CS exposure (Szostak et al., 2020). As oxidative stress is an unifying mechanism for many cardiovascular diseases risk factors (Madamanchi et al., 2005), we might lack one of the contributing factors for developing accelerated atherosclerotic plaques in ApoE^−/−^ mice exposed to CS under NOEC conditions.

Furthermore, other pro‐atherosclerotic factors, such as increased plasma triglycerides levels and body weight gain over time, were not observed in the CS‐exposed NOEC group compared with the WBEC group. In the present study, a pronounced reduction in the levels of all triglyceride classes (i.e., chylomicron, VLDL, and HDL triglycerides) was observed in the CS‐exposed NOEC group, whereas only a reduction in LDL triglyceride levels was observed in the plasma of mice exposed to CS in WBECs. Interestingly, we observed that total, VLDL, and LDL triglyceride levels were reduced in the plasma of the Sham NOEC group compared with the Sham WBEC group. No reduction in triglyceride levels was observed in CS‐exposed ApoE^−/−^ mice in WBECs in previous studies (Phillips et al., 2016; Phillips et al., 2019). However, a reduction in plasma triglyceride levels following exposure to CS or nicotine‐containing test aerosols was observed in previous 28‐ or 90‐day NOEC studies in rats (Phillips, Esposito, et al., 2015; Phillips et al., 2017; Vanscheeuwijck et al., 2002) but not in NO‐exposed C57BL/6 mice (Tsuji et al., 2015). Thus, the mouse strain and their stress status in an NO exposure might affect plasma triglyceride and cholesterol levels. Indeed, in nonfasted rats, triglyceride levels, but not total cholesterol levels, decreased in response to acute immobilization stress (Hershock & Vogel, 1989). Others have reported that chronic stress reduces the levels of triglycerides and total cholesterol, among other parameters, in rats (de Oliveira et al., 2014). Both acute and chronic immobilization stress decreased the plasma triacylglycerol concentration in another study, as reflected by the reduction in the number of VLDL particles in rats (Ricart‐Jane et al., 2002).

Additionally, the lower body weight of mice exposed to fresh air or CS in the NOEC compared with that of mice exposed in the WBEC is an indicator that the mice in the NOEC were more stressed than the WB‐exposed mice (Curtin et al., 2004; Jeong et al., 2013; Van Eijl et al., 2006). The restraint in the NO exposure tubes, in combination with the high plasma nicotine concentration, is thought to have contributed to the higher incidence of tremors in the NO CS‐exposed mice. In comparison with C57BL/6 mice exposed in NOECs under a similar exposure regimen (Tsuji et al., 2015), the ApoE^−/−^ mice appeared more sensitive to restraint and stress from CS exposure. A higher sensitivity to CS effects in WB exposed ApoE^−/−^ mice than in the WB exposed C57BL/6 mice was also observed in our previous inhalation studies (Phillips et al., 2016; 2019).

A difference in the inflammatory response between WB and NO CS‐exposed mice was noted in the white blood cell count. In the NO‐exposed mice, lower numbers of total white blood cells were observed following exposure to CS than after Sham exposure; this decrease was mainly driven by a decrease in lymphocyte count. Reduction in white blood cell and lymphocyte counts were also observed in a previous C57BL/6 mouse CS exposure study using a NOEC (Tsuji et al., 2015). Although we did not observe exposure‐related effects on total and differential white blood cell counts in a previous mouse study on ApoE^−/−^ mice exposed to CS in WBECs (Phillips et al., 2016), we did observe lower lymphocyte counts in rats exposed to nicotine‐containing aerosol in NOECs (Phillips, Esposito, et al., 2015). Therefore, the reduction in white blood cell count might be due to a combination of stress induced by CS exposure and by the stress of restraint during NO exposure. Stressful conditions are known to alter the circulating leukocyte counts (Everds et al., 2013). The lower blood lymphocyte counts in the CS NOEC group represents likely a stress‐related immune change due to the restraint stress in the NOEC. To note, lymphocytes have been shown to have an important role in early pathogenesis of atherosclerotic lesions (Song et al., 2001).

Immune response‐associated signaling pathways were only found statistical significant in the heart ventricle of CS‐exposed WBEC group. CS exposure in WBEC caused downregulation of leukocyte extravasation, integrin, IL‐8, and IL‐1 signaling. A similar downregulation of inflammatory processes in transcriptomics analysis was observed in our previous ApoE^−/−^ study in WBEC after 6 months (Szostak et al., 2017) or after 4 months of CS exposure (Szostak et al., 2020), but not in the NOEC group in this study.

It is tempting to speculate that the higher stress response in CS‐exposed mice in the NOEC as indicated by body weight decrease, lower total plasma triglyceride, and lower blood lymphocyte counts compared with WB exposed mice might attenuate a cardiovascular response.

The absence of obvious changes in red blood cell parameters in mice exposed to CS in NOECs was unexpected, because the test atmospheres in both NOECs and WBECs contained high levels of CO, which led to the formation of COHb, representing reduced oxygen transportation capability that leads to hypoxia. Hypoxia is known to stimulate the production of erythropoietin, a factor that stimulates erythropoiesis, which ultimately leads to the production of more red blood cells and hemoglobin. The changes observed in our study in the WBEC group were consistent with other reports from CS WB inhalation studies in mice that showed increased red blood cell counts and hemoglobin concentrations in C57BL/6 and ApoE^−/−^ mice as a consequence of the high CO concentrations in CS (Phillips et al., 2016; 2019; Phillips, Veljkovic, et al., 2015). The lack of strong erythropoiesis response might be specific for mice in NO exposure. Tsuji et al. reported only subtle increase in hemoglobin concentrations but no increase in red blood cell counts in C57BL/6 mice after 26 and 52 weeks of CS NO exposure (Tsuji et al., 2015).

## 5 SUMMARY AND CONCLUSION

In our study, the CS‐induced respiratory disease endpoints were confirmed in both exposure systems. NO exposure to CS resulted in significantly stronger effects than WB exposure, as indicated by epithelial degeneration and ulceration in nasal epithelia, lung inflammation, and more prominent molecular dysregulation in the respiratory system.

Atherosclerotic plaques acceleration following CS exposure was found only in WBEC. The significant delay in body weight gain after CS exposure and the additional weight loss resulting from NO exposure were indicative of a higher stress response in NO than in WB exposed mice. Additionally, the reduction in triglyceride levels that further declined after CS exposure and the reduction in blood leukocyte/lymphocyte counts after CS exposure in NOEC may point to a higher stress response in the NO‐exposed mice that could lead potentially to an inhibition of atherosclerosis progression. Additionally, NO exposure to CS did not increase erythropoiesis in spite of high COHb values.

Analysis of selected aerosol constituents at matching TPM concentrations indicated differences in CS composition between the EC types that are in part due to the different thermodynamic state of the CS aerosol mixture and different extent of deposition losses along the chamber design which also might contribute to the differences in biological effects. Further differences were determined in the uptake of CS constituents due to the functional differences of the two systems, including additional uptake of, for example, nicotine via noninhalation routes. It would be interesting, in future studies, to elucidate which test atmosphere alterations contribute to the different disease effects.

Overall, the CS exposure regimen and length of the exposure phase in NOECs will need to be further optimized to avoid atypical degenerative effects in the upper respiratory tract while increasing the magnitude of the CS‐accelerated cardiovascular effects in the combined ApoE^−/−^ disease model.

## CONFLICT OF INTEREST

All authors, except WKS, are employees of PMI R&D; WKS was contracted and paid by PMI.

## FUNDING INFORMATION

The work reported in this publication was funded solely by Philip Morris International (PMI).

## Supporting information


**Data S1.** Supporting Information

**Figure S1.** Statistically significant key findings. A) Aerosol generation and uptake. B) Respiratory effects. C) Non‐respiratory effects. Only selected findings are shown. The comparisons are indicated. The triangle indicates statistical significance of raw p < 0.05. Green color indicates a significant lower effect and red color indicates a significant higher effect as per indicated comparison. In summary, aerosol constituent concentrations and uptake into the blood was higher within the CS NOEC group, but nicotine metabolites in the urine were lower in the CS NOEC than in the CS WBEC group. Respiratory findings in the nose were generally higher in the CS NOEC group than in the CS WBEC, whereas findings in the lung histopathology were similar between CS NOEC and CS WBEC groups. Lung volume measurements and finding in the BALF were higher in the CS NOEC group than in the CS WBEC. Non‐respiratory effects were predominantly decreased in in the CS NOEC group than in the CS WBEC. 3R4F, reference cigarette; BALF, bronchoalveolar lavage fluid; CEMA, 2‐cyanoethyl‐mercapturic acid; COHb, carboxyhemoglobin; NNAL, 4‐(methylnitrosamino)‐1‐(3‐pyridyl)‐1‐butanol; NOEC, nose‐only exposure chamber; ns, not significant; TPM, total particulate matter; WBEC, whole‐body exposure chamber. Additional endpoint information as well as data visualizations are available on the INTERVALS platform at https://doi.org/10.26126/intervals.fl34h3.1 *.*

**Figure S2.** Computational fluid dynamics modeling. A) Left: Flow streamlines inside the inner plenum of a nose‐only exposure chamber (NOEC) (CH‐Technology). Right: Particle number density profiles inside the exposure tube of the NOEC system for particles with diameters of 0.05, 0.50, and 4.50 μm. B) Left: Flow streamlines inside the whole‐body exposure chamber. Right: Particle number density profiles on a surface 2 cm from the bottom walls of the box for particles with diameters of 0.05, 0.50, and 4.50 μm.

**Figure S3.** Comparison of differential gene expression in nasal epithelium and differential protein expression in lung tissues between the present and a previous study. A) Comparison of differential gene expression in nasal epithelium in the present and a previous study (Phillips, 2019). B) Comparison of differential proteome expression in lung tissues in the present and a previous study (Phillips, 2019). The columns show the 3R4F contrasts for the current study and the rows the 3R4F contrasts for the previous study (Phillips, 2019), with the number of differentially expressed genes (A) or proteins (B) shown in the margins. The correlation coefficient between the contrasts is color‐coded, and the number of shared differentially expressed proteins is indicated in the cells of the matrix. The percentage of shared differentially expressed genes (A) or proteins (B) with the same direction of change is shown as pie charts. Green asterisk indicates significance of the observed overlap of the differentially expressed proteins (Fisher's test p value < 0.05). 3R4F, reference cigarette; FC, fold change; mo, month, NOEC, nose‐only exposure chamber; WBEC, whole‐body exposure chamber.

**Table S1.** Nominal concentrations, achieved concentrations, and yields of TPM, nicotine, and carbonyls in the exposure chambers.

**Table S2.** Histopathological findings in the nose (level 1, 2 and 4).

**Table S3**. Histopathological findings (incidences) in the nose and lung.

**Table S4**. Inflammatory mediators in BALF.

## Data Availability

The data that support the findings of this study are openly available at https://doi.org/10.26126/intervals.fl34h3.1.
