# Supplementary material for: Impact of whole‐body versus nose‐only inhalation exposure systems on systemic, respiratory, and cardiovascular endpoints in a 2‐month cigarette smoke exposure study in the ApoE−/− mouse model
Source: J Appl Toxicol. 2021 Apr 6;41(10):1598–619. doi: 10.1002/jat.4149 (PMC8519037; doi:10.1002/jat.4149)
Supplement: Supplementary file 1 — Data S1. Supporting Information [file JAT-41-1598-s002.pdf]

Impact of whole-body versus nose-only inhalation exposure systems on systemic, respiratory, and cardiovascular endpoints in a 2-month cigarette smoke exposure study in the ApoE$^{-/-}$ mouse model

Ulrike Kogel [a]*, Ee Tsin Wong [b]*, Justyna Szostak [a], Wei Teck Tan [b], Francesco Lucci [a], Patrice Leroy [a], Bjoern Titz [a], Yang Xiang [a], Tiffany Low [b], Sin Kei Wong [b], Emmanuel Guedj [a], Nikolai V. Ivanov [a], Walter K. Schlage [c], Manuel C. Peitsch [a], Arkadiusz Kuczaj [a], Patrick Vanscheeuwijck [a], Julia Hoeng [a]

[a] Philip Morris International Research and Development, Philip Morris Products S.A., Quai Jeanrenaud 5, 2000 Neuchatel, Switzerland
[b] Philip Morris International Research Laboratories Pte. Ltd., Science Park II, Singapore
[c] Biology Consultant, Max-Baermann-Str. 21, 51429, Bergisch Gladbach, Germany

*equal contribution, shared co-first authorship

Corresponding author:
Julia Hoeng, PhD
E-mail: julia.hoeng@pmi.com
Tel: +41 (58) 242 2214
Fax: +41 (58) 242 2811

Supplementary

Computational fluid dynamics (CFD) modeling

Aim: The aim of applying CFD simulations was to verify the contribution of exposure chamber geometries, physical aerosol properties, and flow conditions to the potential significant deposition losses inside these systems that could lead to aerosol size-dependent filtration and selective aerosol inhalation, causing particular imbalance in inhaled aerosol ratios between these systems. Understanding the complexities of the differences between the two types of systems/inhalations (NO and WB) that cannot be computationally tackled in detail, we concentrated our simulation studies on finding potential discrepancies arising from inhalation of non-evolving aerosols of various particle sizes, investigating flow and aerosol distribution uniformity, and possible size-selective aerosol sampling inside these two chambers.

Materials and Methods: Flow and aerosol concentration uniformity within the exposure chambers were investigated by using AeroSolved, a recently developed and publicly available CFD simulation platform (http://www.aerosolved.com). The AeroSolved code was developed to

study aerosol dynamics from generation through evolution, transport, and deposition. The platform is based on OpenFOAM, a well-established open-source CFD framework. The transport and deposition of the aerosol are mathematically described by a system of multiphase transport equations by using an Eulerian–Eulerian approach, where gas and aerosol particles are modeled as continuous fields (Frederix, 2016a; Frederix, 2016b; Winkelmann et al., 2018).

The geometrical complexity of the NOEC and WBEC systems required several assumptions to be made for modeling flow uniformity. An aerosol consisting of polystyrene latex particles with different geometrical diameters, ranging from 0.02 to 4.5 µm, was analyzed. (Note that the diameter here is equivalent to the MMAD of monodisperse aerosol [GSD = 1], neglecting small particle density correction). All aging effects of the aerosol—such as constituent and phase repartitioning by condensation/evaporation and chemical reactions—were neglected. Aerosol transport was modeled by using a particle size-dependent drift model. Brownian diffusion was modeled with a Stokes–Einstein diffusion coefficient. Deposition due to sedimentation and impaction was also included (Frederix, 2016).

In the NOEC system, only the inner plenum region, including the exposure channels at each exposure tube, was simulated, neglecting the effect of the outer plenum. A more detailed analysis of the exposure tube was also performed to quantify the effect of aerosol sampling at the nostril of the animal. The aerosol flow rate at the NOEC inlet was set to 0.5 L/min at each exposure tube. In the WBEC, only one row of three exposure cages was taken as the computational simulation/geometrical domain, assuming similarity among all rows. The effects of animal movement on the flow inside the cages and the effect of extra components, such as the cage cover, water bottles, and bedding materials, were neglected. Aerosol was delivered to each exposure cage at a jet flow rate of 5.54 L/min. The jets were rotated by an angle of 45° with respect to the main exposure cage axes to limit the aerosol losses due to jet impaction on the front door. On the top plane of the computational domain, the flow was set to 50 L/min, equivalent to the flow experienced in the 4th row from the top of the WBEC. The aerosol concentrations on the top plane were set equal to the aerosol inlet delivery jet concentrations. All flows were analyzed in a steady-state condition.

Results: Inside the inner plenum of the NOEC, the inlet aerosol jet was observed to create a steady recirculation zone (Supplementary Figure 2*A). While the average flow in the top–down NOEC configuration is downward, the recirculation produces a region with an upward flow close to the inner plenum wall. A direct consequence of this steady flow pattern is a flow variability at the exposure tube inlets (called “ports” here, for simplicity) of up to ±30% of the flow average. The ports in the top tiers have a lower flow rate than those in in the bottom tiers. Aerosol deliveries at each port were not correlated with the flow variability, because, for each particle diameter, the concentration variability between the ports was at most 2%. However, for particles >3 µm in size, aerosol concentration losses in the order of 10% were predicted at the ports, compared with the concentrations at the chamber inlet. For these particles, aerosol separation due to gravity settling was observed close to the top wall of the port. Furthermore, under the present simulation conditions, small aerosol particle size-selective sampling effects (<2%) were observed at the nostril level.

In WBECs, multiple recirculation zones were created by the aerosol inlet jets. Flow redistribution was observed not only between different exposure cages, where the aerosol is transported from one cage to the other, but also within each box, where the main flow streams are separated into smaller flow vortices (Supplementary Figure 2*B). Moreover, the asymmetric jet orientations caused differences in the flow between cages present in the same row. In

Supplementary Figure 1B, cages 1 and 2 had more irregular flow structures, while cage 3 had a more uniform flow. While cages 1 and 2 received most of their flow directly from the inlet jet, the flow entering the cage 3 came from the neighboring cages. The aerosol distribution in the plane 2 cm over the cage bottom showed some localized regions of lower aerosol concentration.

Discussion*:* One of the main factors influencing aerosol delivery in the two chambers, apart from the various routes of deposition, is related to animal movement. In the NOEC system, the animals are constrained, and the aerosol losses were estimated to be within ±10% for inhalable aerosols (particle diameter <3 μm). In WBECs, our analysis showed only a small aerosol non-uniformity at the bottom of the chamber, but did not exclude some regions of less concentrated aerosol linked to the flow structures (Supplementary Figure 2*B). Animal movements could potentially change the flow within the cage, and the animals might adapt to breathing aerosol from the low concentration spots, increasing the non-uniformity effects in the cages. Overall, in both chambers, when only inhalable aerosols were considered, the analysis showed the variability of exposure to an aerosol be to within 10% (Yeh et al., 1990), confirming the suitability of the chambers for inhalation toxicology studies. Both chambers still entail some variability that can affect animal exposure or comfort; thus, animal or cage rotation within each chamber is recommended to increase the statistical uniformity of endpoints measurements.

References Supplement

Frederix, E.M.A. (2016a). Eulerian modeling of aerosol dynamics (University of Twente).

Frederix, E.M.A. (2016b). Eulerian modeling of aerosol dynamics. (https://doi.org/10.3990/1.9789036542289, University of Twente).

Winkelmann, C., Kuczaj, A.K., Nordlund, M., and Geurts, B.J. (2018). Simulation of aerosol formation due to rapid cooling of multispecies vapors. Journal of Engineering Mathematics *108*, 171-196.

Yeh, H., Snipes, M., Eidson, A., Hobbs, C., and Henry, M. (1990). Comparative evaluation of nose-only versus whole-body inhalation exposures for rats—Aerosol characteristics and lung deposition. Inhalation Toxicology *2*, 205-221.

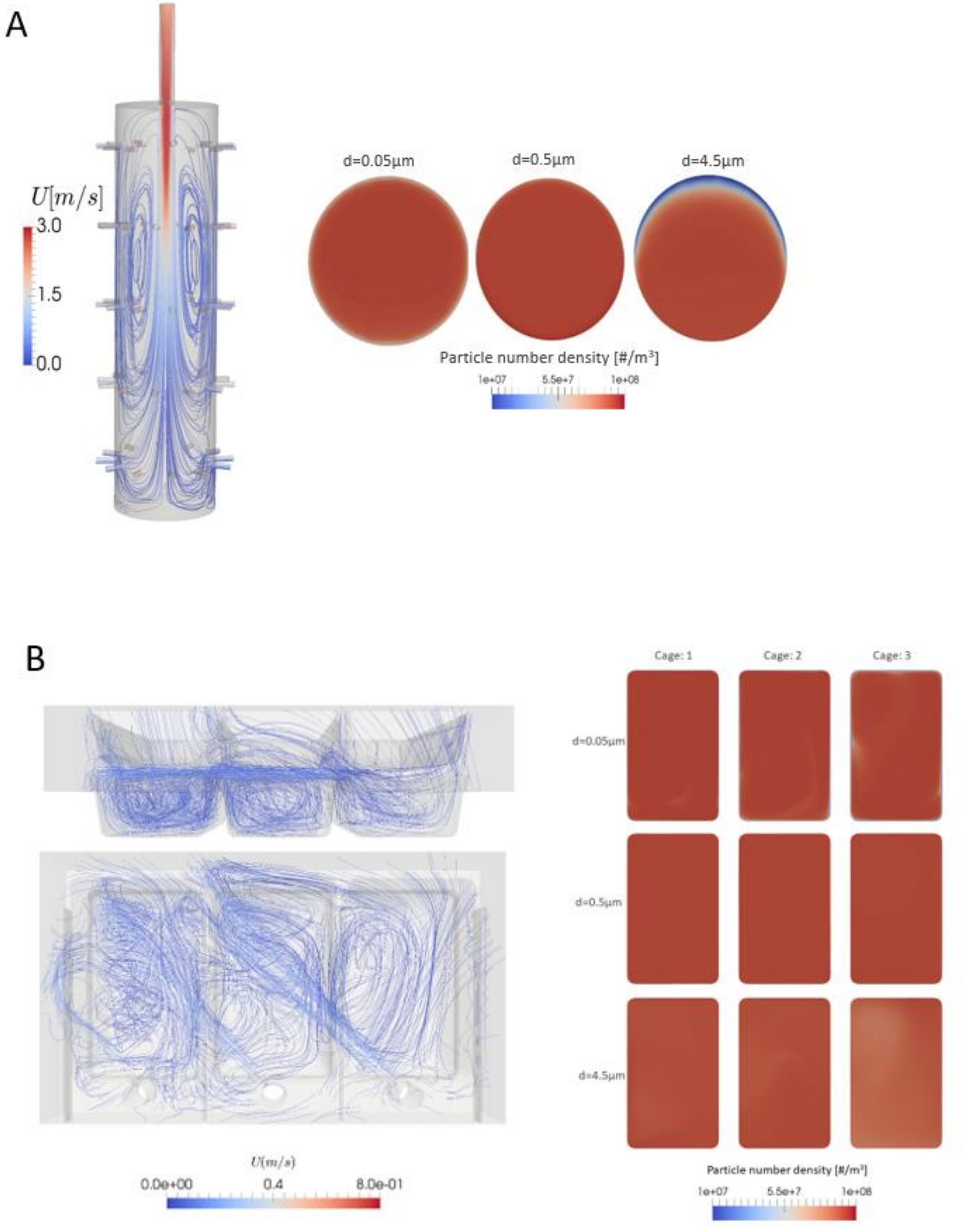

Supplementary Figure 2*. Computational fluid dynamics modeling. *A) Left: Flow streamlines inside the inner plenum of a nose-only exposure chamber (NOEC) (CH-Technology). Right: Particle number density profiles inside the exposure tube of the NOEC system for particles with diameters of 0.05, 0.50, and 4.50 µm. B) Left: Flow streamlines inside the whole-body exposure chamber. Right: Particle number density profiles on a surface 2 cm from the bottom walls of the box for particles with diameters of 0.05, 0.50, and 4.50 µm.*

* number 2 refers to the numbering in the main article.
