# Supplementary material for: Impact of whole‐body versus nose‐only inhalation exposure systems on systemic, respiratory, and cardiovascular endpoints in a 2‐month cigarette smoke exposure study in the ApoE−/− mouse model
Source: J Appl Toxicol. 2021 Apr 6;41(10):1598–619. doi: 10.1002/jat.4149 (PMC8519037; doi:10.1002/jat.4149)
Supplement: Supplementary file 2 — Figure S1. Statistically significant key findings. A) Aerosol generation and uptake. B) Respiratory effects. C) Non‐respiratory effects. Only selected findings are shown. The comparisons are indicated. The triangle indicates statistical significance of raw p < 0.05. Green color indicates a significant lower effect and red color indicates a significant higher effect as per indicated comparison. In summary, aerosol constituent concentrations and uptake into the blood was higher within the CS NOEC group, but nicotine metabolites in the urine were lower in the CS NOEC than in the CS WBEC group. Respiratory findings in the nose were generally higher in the CS NOEC group than in the CS WBEC, whereas findings in the lung histopathology were similar between CS NOEC and CS WBEC groups. Lung volume measurements and finding in the BALF were higher in the CS NOEC group than in the CS WBEC. Non‐respiratory effects were predominantly decreased in in the CS NOEC group than in the CS WBEC. 3R4F, reference cigarette; BALF, bronchoalveolar lavage fluid; CEMA, 2‐cyanoethyl‐mercapturic acid; COHb, carboxyhemoglobin; NNAL, 4‐(methylnitrosamino)‐1‐(3‐pyridyl)‐1‐butanol; NOEC, nose‐only exposure chamber; ns, not significant; TPM, total particulate matter; WBEC, whole‐body exposure chamber. Additional endpoint information as well as data visualizations are available on the INTERVALS platform at https://doi.org/10.26126/intervals.fl34h3.1 . [file JAT-41-1598-s004.pdf]

Supplementary Figure 1. Statistically significant key findings.

A

Aerosol generation and uptake

			3R4F WBEC vs Sham WBEC	3R4F NOEC vs Sham NOEC	Sham NOEC vs Sham WBEC	3R4F NOEC vs 3R4F WBEC
Aerosol generation	Exposure Chamber	Carbon monoxide (ppm)	▲	▲	▲	▲
		Formaldehyde (µg/L)	▲	▲	▼	▲
		Nicotine (µg/L)	▲	▲	ns	▲
		TPM (µg/L)	▲	▲	▼	ns
Aerosol uptake and exposure	Marker of Exposure in blood	COHb (%)	▲	▲	ns	▲
	Marker of Exposure in plasma	Cotinine (ng/mL)	▲	▲	ns	▲
		Nicotine (ng/mL)	▲	▲	ns	ns
	Marker of exposure in urine (rel. creatinine)	CEMA (µg/mol)	▲	▲	▼	▲
		Total NNAL (ng/mol)	▲	▲	ns	ns
	Nicotine metabolites in urine (rel. creatinine)	Cotinine	▲	▲	ns	▼
		Nicotine	▲	▲	ns	▼
		Norcotinine	▲	▲	ns	▼

▲ statistically significant upregulated (raw $p < 0.05$)
▼ statistically significant downregulated (raw $p < 0.05$)
ns not statistically significant

B

Respiratory effects (nose, lung)

			3R4F WBEC vs Sham WBEC	3R4F NOEC vs Sham NOEC	Sham NOEC vs Sham WBEC	3R4F NOEC vs 3R4F WBEC
Histopathology	Nose level 1	Respiratory epithelium, degeneration (Score)	ns	▲	ns	▲
		Respiratory epithelium, hyperplasia (Score)	▲	▲	ns	ns
		Respiratory epithelium, squamous epithelial metaplasia (Score)	▲	▲	ns	▲
		Respiratory epithelium, ulceration (Score)	ns	▲	ns	▲
	Nose level 2	Olfactory epithelium, atrophy (Score)	ns	▲	ns	ns
		Respiratory epithelium, degeneration (Score)	ns	▲	ns	▲
		Respiratory epithelium, hyperplasia (Score)	ns	▲	ns	▲
		Respiratory epithelium, squamous epithelial metaplasia (Score)	ns	▲	ns	▲
		Respiratory epithelium, ulceration (Score)	ns	▲	ns	▲
	Nose level 4	Olfactory epithelium, atrophy (Score)	ns	▲	ns	▲
		Olfactory region, lumen, amorphous eosinophilic material (Score)	ns	▲	ns	▲
	Left lung	Alveolar lumen, unpigmented macrophages (Score)	▲	▲	ns	ns
		Alveolar lumen, yellow pigmented macrophages (Score)	▲	▲	ns	ns
		Perivascular mono-nuclear inflammatory cells (Score)	▲	▲	ns	ns
Gross pathology	Absolute weight after exangination	Lung weight (g)	▲	▲	ns	ns
		Lung volume (g)	ns	▲	ns	ns
BALF	Free lung cells	Total cell count	▲	▲	ns	▲
Lung function	Forced Expiratory Volume Analyzer	Forced expiratory volume at 0.05 seconds (mL)	▲	▲	ns	▲
		Forced expiratory volume at 0.20 seconds (mL)	▲	▲	ns	▲
		Forced vital capacity (mL)	▲	▲	ns	▲
	Inspiratory Capacity Analyzer	Inspiratory capacity (mL)	▲	▲	ns	▲

▲ statistically significant upregulated (raw $p < 0.05$)
ns not statistically significant

C

Non-respiratory effects (liver, heart, blood)

			3R4F WBEC vs Sham WBEC	3R4F NOEC vs Sham NOEC	Sham NOEC vs Sham WBEC	3R4F NOEC vs 3R4F WBEC
Gross pathology	Absolute weight after exangination	Body weight (g)	ns	▼	▼	▼
		Liver weight (g)	ns	▼	ns	▼
	Aortic Arch	Plaque area (mm²)	▲	ns	ns	ns
		Plaque area relative to aorta area (%)	ns	ns	ns	ns
Hematology	Platelet	Platelet (10^3/µL)	▼	ns	ns	▲
	Red blood cells	Erythrocyte (10^6/µL)	▲	ns	ns	▼
		Hematocrit (L/L)	▲	ns	ns	▼
		Hemoglobin (g/L)	▲	ns	ns	▼
	White blood cell count	Total cell counts (10^3/µL)	▲	▼	ns	▼
	White blood cell differential absolute	Lymphocyte counts (10^3/µL)	ns	▼	ns	▼
		Neutrophil counts (10^3/µL)	▲	ns	▲	ns
	White blood cell differential relative	Lymphocyte counts (relative) (%)	ns	ns	▼	▼
		Neutrophil counts (relative) (%)	ns	ns	▲	▲
Lipid profile	Plasma cholesterol profile	Total cholesterol (mg/dL)	ns	▲	ns	ns
	Plasma triglycerides profile	Total triglycerides (mg/dL)	ns	▼	▼	▼
Clinical chemistry	Proteins	Albumin (g/L)	▲	▲	▲	ns

▲ statistically significant upregulated (raw $p < 0.05$)
▼ statistically significant downregulated (raw $p < 0.05$)
ns not statistically significant

Supplementary Figure 1. Statistically significant key findings. A) Aerosol generation and uptake. B) Respiratory effects. C) Supplementary Figure 1. Statistically significant key findings. Non-respiratory effects. Only selected findings are shown. The comparisons are indicated. The triangle indicates statistical significance of raw $p < 0.05$. Green color indicates a significant lower effect and red color indicates a significant higher effect as per indicated comparison. In summary, aerosol constituent concentrations and nicotine uptake into the blood were higher within the CS NOEC group, but nicotine metabolites in the urine were lower in the CS NOEC than in the CS WBEC group. Respiratory findings in the nose were generally higher in the CS NOEC group than in the CS WBEC, whereas findings in the lung histopathology were similar between CS NOEC and CS WBEC groups. Lung volume measurements and finding in the BALF were higher in the CS NOEC group than in the CS WBEC. Non-respiratory effects were predominantly decreased in in the CS NOEC group than in the CS WBEC. 3R4F, reference cigarette; BALF, bronchoalveolar lavage fluid; CEMA, 2-cyanoethyl-mercapturic acid; COHb, carboxyhemoglobin; NNAL, 4-(methylnitrosamino)-1-(3-pyridyl)-1-butanol; NOEC, nose-only exposure chamber; ns, not significant; TPM, total particulate matter; WBEC, whole-body exposure chamber. Additional endpoint information as well as data visualizations are available on the INTERVALS platform at https://doi.org/10.26126/intervals.fl34h3.1.
