# Supplementary material for: Impact of whole‐body versus nose‐only inhalation exposure systems on systemic, respiratory, and cardiovascular endpoints in a 2‐month cigarette smoke exposure study in the ApoE−/− mouse model
Source: J Appl Toxicol. 2021 Apr 6;41(10):1598–619. doi: 10.1002/jat.4149 (PMC8519037; doi:10.1002/jat.4149)
Supplement: Supplementary file 3 — Figure S2. Computational fluid dynamics modeling. A) Left: Flow streamlines inside the inner plenum of a nose‐only exposure chamber (NOEC) (CH‐Technology). Right: Particle number density profiles inside the exposure tube of the NOEC system for particles with diameters of 0.05, 0.50, and 4.50 μm. B) Left: Flow streamlines inside the whole‐body exposure chamber. Right: Particle number density profiles on a surface 2 cm from the bottom walls of the box for particles with diameters of 0.05, 0.50, and 4.50 μm. [file JAT-41-1598-s005.pdf]

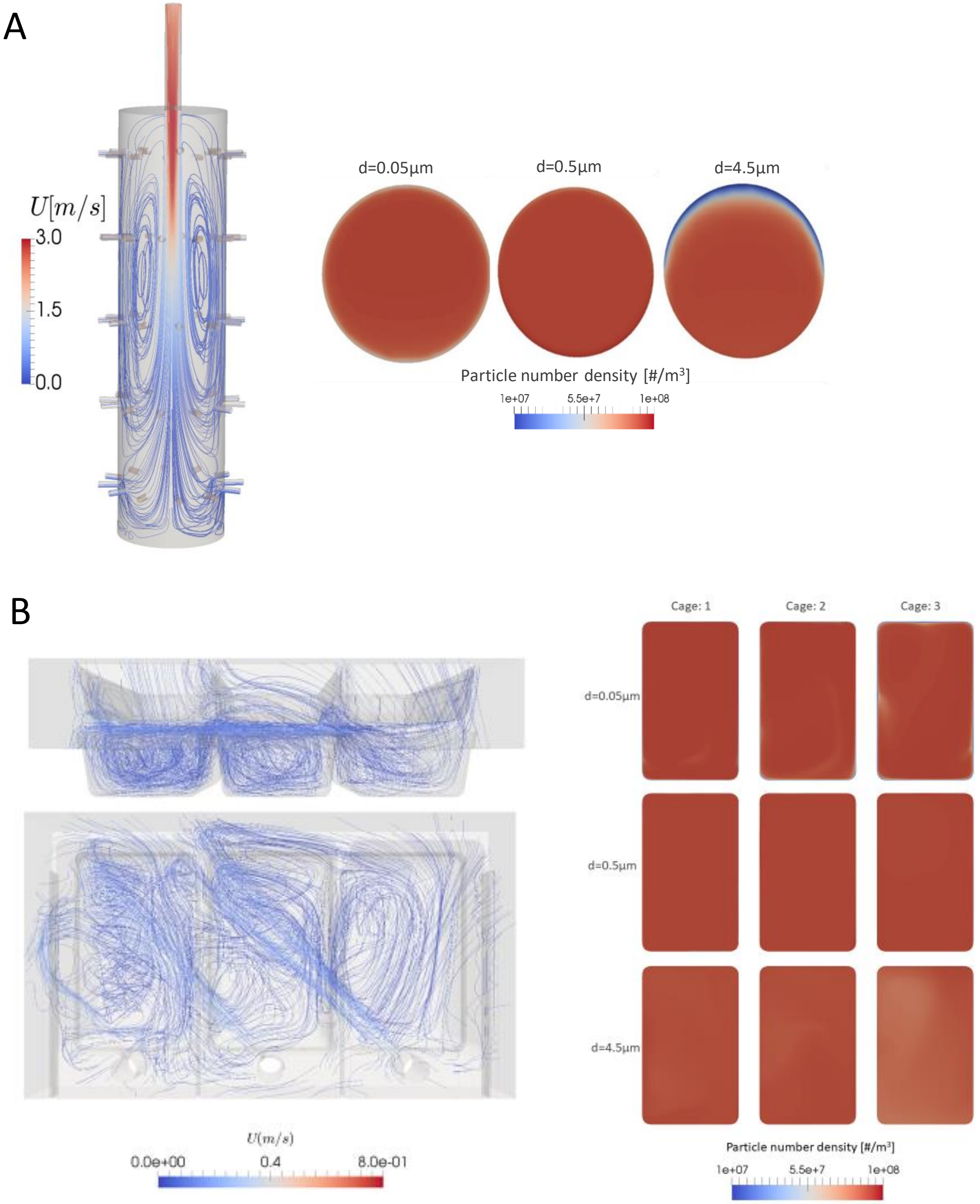

Supplementary Figure 2. Computational fluid dynamics modeling. ***A)*** *Left: Flow streamlines inside the inner plenum of a nose-only exposure chamber (NOEC) (CH-Technology). Right: Particle number density profiles inside the exposure tube of the NOEC system for particles with diameters of 0.05, 0.50, and 4.50 μm.* ***B)*** *Left: Flow streamlines inside the whole-body exposure chamber. Right: Particle number density profiles on a surface 2 cm from the bottom walls of the box for particles with diameters of 0.05, 0.50, and 4.50 μm.*
