# Supplementary material for: Impact of whole‐body versus nose‐only inhalation exposure systems on systemic, respiratory, and cardiovascular endpoints in a 2‐month cigarette smoke exposure study in the ApoE−/− mouse model
Source: J Appl Toxicol. 2021 Apr 6;41(10):1598–619. doi: 10.1002/jat.4149 (PMC8519037; doi:10.1002/jat.4149)
Supplement: Supplementary file 4 — Figure S3. Comparison of differential gene expression in nasal epithelium and differential protein expression in lung tissues between the present and a previous study. A) Comparison of differential gene expression in nasal epithelium in the present and a previous study (Phillips, 2019). B) Comparison of differential proteome expression in lung tissues in the present and a previous study (Phillips, 2019). The columns show the 3R4F contrasts for the current study and the rows the 3R4F contrasts for the previous study (Phillips, 2019), with the number of differentially expressed genes (A) or proteins (B) shown in the margins. The correlation coefficient between the contrasts is color‐coded, and the number of shared differentially expressed proteins is indicated in the cells of the matrix. The percentage of shared differentially expressed genes (A) or proteins (B) with the same direction of change is shown as pie charts. Green asterisk indicates significance of the observed overlap of the differentially expressed proteins (Fisher's test p value < 0.05). 3R4F, reference cigarette; FC, fold change; mo, month, NOEC, nose‐only exposure chamber; WBEC, whole‐body exposure chamber. [file JAT-41-1598-s007.pdf]

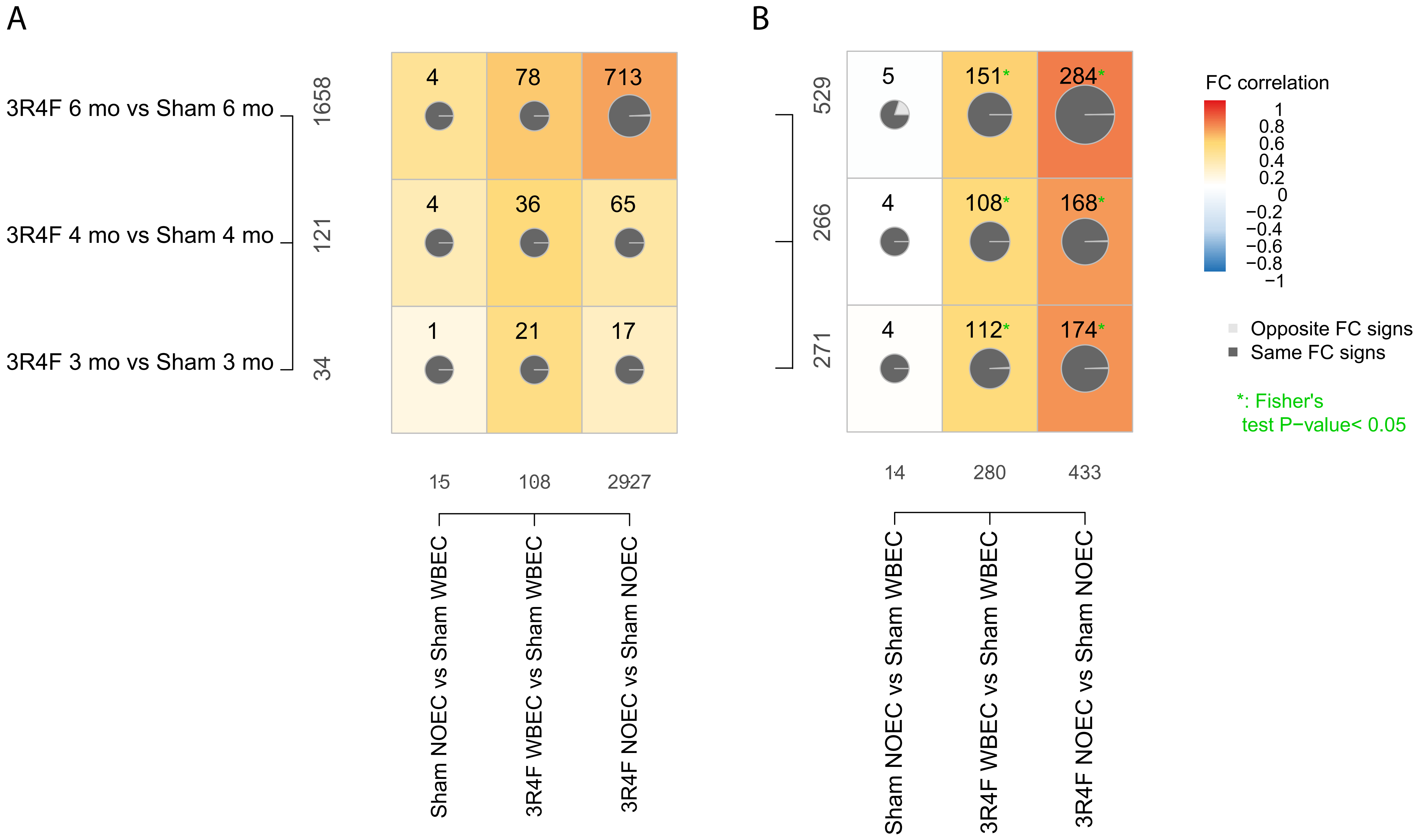

Supplementary Figure 3. Comparison of differential gene expression in nasal epithelium and differential protein expression in lung tissues between the present and a previous study. A) Comparison of differential gene expression in nasal epithelium in the present and a previous study (Phillips, 2019). B) Comparison of differential proteome expression in lung tissues in the present and a previous study (Phillips, 2019). The columns show the 3R4F contrasts for the current study and the rows the 3R4F contrasts for the previous study (Phillips, 2019), with the number of differentially expressed genes (A) or proteins (B) shown in the margins. The correlation coefficient between the contrasts is color-coded, and the number of shared differentially expressed proteins is indicated in the cells of the matrix. The percentage of shared differentially expressed genes (A) or proteins (B) with the same direction of change is shown as pie charts. Green asterisk indicates significance of the observed overlap of the differentially expressed proteins (Fisher's test p value < 0.05). 3R4F, reference cigarette; FC, fold change; mo, month, NOEC, nose-only exposure chamber; WBEC, whole-body exposure chamber.
