# Supplementary material for: Impact of whole‐body versus nose‐only inhalation exposure systems on systemic, respiratory, and cardiovascular endpoints in a 2‐month cigarette smoke exposure study in the ApoE−/− mouse model
Source: J Appl Toxicol. 2021 Apr 6;41(10):1598–619. doi: 10.1002/jat.4149 (PMC8519037; doi:10.1002/jat.4149)
Supplement: Supplementary file 5 — Table S1. Nominal concentrations, achieved concentrations, and yields of TPM, nicotine, and carbonyls in the exposure chambers. [file JAT-41-1598-s006.pdf]

Supplementary Table 1. Nominal concentrations, achieved concentrations, and yields of TPM, nicotine, and carbonyls in the exposure chambers.

	Nominal concentration generated					Achieved concentration in chamber					Yields				
Chamber	TPM (µg/L)	Nicotine (µg/L)	Acetaldehyde (µg/L)	Acrolein (µg/L)	Formaldehyde (µg/L)	TPM (µg/L)	Nicotine (µg/L)	Acetaldehyde (µg/L)	Acrolein (µg/L)	Formaldehyde (µg/L)	TPM (%)	Nicotine (%)	Acetaldehyde (%)	Acrolein (%)	Formaldehyde (%)
3R4F WBEC	894.5	45.1	36.4	3.7	16	538.6	30.5	34.4	2.9	0.7	60.2	67.6	94.4	79.7	43.9
3R4F NOEC	967	48.7	39.4	4	18	545	39.1	34.4	3	0.9	56.4	80.3	87.4	75.7	50.4

Nominal concentrations of TPM and nicotine were calculated based on a historical puff count of 10.5 puffs/cig when the 3R4F cigarette is smoked under the Health Canada intense smoking protocol in an SM2000 rotary smoking machine; and based on TPM, nicotine, acetaldehyde, acrolein, and formaldehyde content of 37.7 mg/cig, 1.9 mg/cig, 1534 µg/cig, 155 µg/cig, and 68.1 µg/cig, respectively, in a 3R4F cigarette (Roemer et al., 2012)*; and 30 puffs per smoking machine per minute. First, we calculated the TPM, nicotine, acetaldehyde, acrolein, and formaldehyde content generated per minute by multiplying the number of puffs taken per smoking machine divided by the average number of puffs taken per cigarette and multiplying by the TPM, nicotine, acetaldehyde, acrolein, and formaldehyde content in a 3R4F cigarette. This gives 107.7 mg/min of TPM, 5.4 mg/min of nicotine, 4382.9 µg/min of acetaldehyde, 442.9 µg/min of acrolein, and 194.6 µg/min of formaldehyde generated by a single smoking machine within a minute. By dividing these amounts by the flow rates of the PDSP pumps and dilution flows, we obtain the average TPM, carbonyl and nicotine concentrations. In case of WBECs, this gives 894.5 µg/L of TPM, 45.1 µg/L of nicotine, 36.4 µg/L of acetaldehyde, 3.7 µg/L of acrolein, and 1.6 µg/L of formaldehyde, taking into account that two smoking machines are used for generating smoke. In case of NOECs, because of partial removal (1.1 L/min) of the smoke by the four-piston pump, only the ratio of (1.6 - 1.1) / 1.6 = 0.3125 was used for subsequent dilution, which gave concentrations of 967.0 µg/L of TPM, 48.7 µg/L of nicotine, 39.4 µg/L of acetaldehyde, 4.0 µg/L of acrolein, and 1.8 µg/L of formaldehyde. 3R4F, reference cigarette; NOEC, nose-only exposure chamber; WBEC, whole-body exposure chamber; TPM, total particulate matter.*
