# Supplementary material for: Impact of whole‐body versus nose‐only inhalation exposure systems on systemic, respiratory, and cardiovascular endpoints in a 2‐month cigarette smoke exposure study in the ApoE−/− mouse model
Source: J Appl Toxicol. 2021 Apr 6;41(10):1598–619. doi: 10.1002/jat.4149 (PMC8519037; doi:10.1002/jat.4149)
Supplement: Supplementary file 6 — Table S2. Histopathological findings in the nose (level 1, 2 and 4). [file JAT-41-1598-s003.pdf]

Supplementary Table 2. Histopathological findings in the nose (level 1, 2 and 4).

		Sham WBEC	3R4F WBEC	Sham NOEC	3R4F NOEC
Nose level 1	Respiratory epithelium, cornification (Score)	**0.00** ± 0.00	**0.00** ± 0.00	**0.00** ± 0.00	**0.40** ± 0.16 **$ &**
	Respiratory epithelium, degeneration (Score)	**0.00** ± 0.00	**0.20** ± 0.13	**0.00** ± 0.00	**1.20** ± 0.29 **$ &**
	Respiratory epithelium, hyperplasia (Score)	**0.20** ± 0.20	**2.70** ± 0.15 **&**	**0.50** ± 0.17	**3.10** ± 0.10 **&**
	Respiratory epithelium, intraepithelial eosinophilic deposits (Score)	**0.20** ± 0.20	**0.00** ± 0.00	**0.00** ± 0.00	**0.00** ± 0.00
	Respiratory epithelium, squamous epithelial metaplasia (Score)	**0.20** ± 0.20	**2.40** ± 0.16 **&**	**0.50** ± 0.17	**3.80** ± 0.13 **$ &**
	Respiratory epithelium, ulceration (Score)	**0.00** ± 0.00	**0.10** ± 0.10	**0.00** ± 0.00	**1.00** ± 0.30 **$ &**
	Respiratory region, lumen, amorphous eosinophilic material (Score)	**0.00** ± 0.00	**0.10** ± 0.10	**0.00** ± 0.00	**0.90** ± 0.28 **$ &**
	Respiratory region, lumen, necrotic cells (Score)	**0.30** ± 0.30	**0.00** ± 0.00	**0.00** ± 0.00	**0.50** ± 0.17 **$ &**
	Respiratory region, lumen, plant material (Score)	**0.00** ± 0.00	**0.00** ± 0.00	**0.00** ± 0.00	**0.10** ± 0.10
Nose level 2	Olfactory epithelium, atrophy (Score)	**0.20** ± 0.20	**0.70** ± 0.42	**0.00** ± 0.00	**2.20** ± 0.51 **&**
	Olfactory epithelium, intraepithelial eosinophilic deposits (Score)	**0.00** ± 0.00	**0.30** ± 0.30	**0.00** ± 0.00	**0.00** ± 0.00
	Olfactory epithelium, lamina propria, loss of nerve bundles (Score)	**0.00** ± 0.00	**0.30** ± 0.30	**0.00** ± 0.00	**2.20** ± 0.68 **$ &**
	Olfactory epithelium, squamous epithelial metaplasia (Score)	**0.00** ± 0.00	**0.20** ± 0.20	**0.00** ± 0.00	**0.20** ± 0.20
	Olfactory region, lumen, amorphous eosinophilic material (Score)	**0.00** ± 0.00	**0.00** ± 0.00	**0.00** ± 0.00	**0.10** ± 0.10
	Respiratory epithelium, degeneration (Score)	**0.00** ± 0.00	**0.00** ± 0.00	**0.00** ± 0.00	**1.30** ± 0.40 **$ &**
	Respiratory epithelium, hyperplasia (Score)	**0.00** ± 0.00	**0.00** ± 0.00	**0.00** ± 0.00	**1.80** ± 0.29 **$ &**
	Respiratory epithelium, intraepithelial eosinophilic deposits (Score)	**0.00** ± 0.00	**0.00** ± 0.00	**0.50** ± 0.27	**0.00** ± 0.00
	Respiratory epithelium, squamous epithelial metaplasia (Score)	**0.00** ± 0.00	**0.00** ± 0.00	**0.00** ± 0.00	**1.60** ± 0.31 **$ &**
	Respiratory epithelium, ulceration (Score)	**0.00** ± 0.00	**0.00** ± 0.00	**0.00** ± 0.00	**1.10** ± 0.35 **$ &**
	Respiratory region, lumen, amorphous eosinophilic material (Score)	**0.00** ± 0.00	**0.00** ± 0.00	**0.00** ± 0.00	**0.80** ± 0.29 **$ &**
	Respiratory region, lumen, necrotic cells (Score)	**0.30** ± 0.30	**0.00** ± 0.00	**0.00** ± 0.00	**0.10** ± 0.10
	Respiratory region, lumen, plant material (Score)	**0.30** ± 0.30	**0.00** ± 0.00	**0.00** ± 0.00	**0.10** ± 0.10
	Respiratory region, submucosal gland, ectasis (Score)	**0.00** ± 0.00	**0.00** ± 0.00	**0.10** ± 0.10	**0.00** ± 0.00
Nose level 4	Olfactory epithelium, atrophy (Score)	**0.00** ± 0.00	**0.10** ± 0.10	**0.00** ± 0.00	**1.60** ± 0.37 **$ &**
	Olfactory epithelium, lamina propria, loss of nerve bundles (Score)	**0.00** ± 0.00	**0.00** ± 0.00	**0.00** ± 0.00	**0.20** ± 0.20
	Olfactory region, lumen, amorphous eosinophilic material (Score)	**0.00** ± 0.00	**0.10** ± 0.10	**0.00** ± 0.00	**2.40** ± 0.56 **$ &**
	Olfactory region, lumen, red blood cells (Score)	**0.10** ± 0.10	**0.00** ± 0.00	**0.00** ± 0.00	**0.00** ± 0.00

Mean: 0.00 – 3.80

Mean severity scores ± standard error of the mean are shown. Higher scores are shown in darker colors. &, statistically significant differences between the test atmosphere (3R4F) and fresh air (Sham) groups (raw $p < 0.05$). #, statistically significant differences between the Sham groups in the NOEC and WBEC (raw $p < 0.05$). $, statistically significant differences between 3R4F groups in the NOEC and WBEC (raw $p < 0.05$). 3R4F, reference cigarette; NOEC, nose-only exposure chamber; WBEC, whole-body exposure chamber.
