# Supplementary material for: Impact of whole‐body versus nose‐only inhalation exposure systems on systemic, respiratory, and cardiovascular endpoints in a 2‐month cigarette smoke exposure study in the ApoE−/− mouse model
Source: J Appl Toxicol. 2021 Apr 6;41(10):1598–619. doi: 10.1002/jat.4149 (PMC8519037; doi:10.1002/jat.4149)
Supplement: Supplementary file 7 — Table S3. Histopathological findings (incidences) in the nose and lung. [file JAT-41-1598-s008.pdf]

Supplementary Table 3. Histopathological findings (incidences) in the nose and lung.

		Sham WBEC				3R4F WBEC					Sham NOEC				3R4F NOEC				
	Severity grading	0	1	2	3	0	1	2	3	4	0	1	2	3	0	1	2	3	4
Nose level 1	Respiratory epithelium, cornification (Score)	10 / 10				10 / 10					10 / 10				6 / 10	4 / 10			
	Respiratory epithelium, degeneration (Score)	10 / 10				8 / 10	2 / 10				10 / 10				3 / 10	2 / 10	5 / 10		
	Respiratory epithelium, hyperplasia (Score)	9 / 10		1 / 10				3 / 10	7 / 10		5 / 10	5 / 10						9 / 10	1 / 10
	Respiratory epithelium, intraepithelial eosinophilic deposits (Score)	9 / 10		1 / 10		10 / 10					10 / 10				10 / 10				
	Respiratory epithelium, squamous epithelial metaplasia (Score)	9 / 10		1 / 10				6 / 10	4 / 10		5 / 10	5 / 10						2 / 10	8 / 10
	Respiratory epithelium, ulceration (Score)	10 / 10				9 / 10	1 / 10				10 / 10				4 / 10	2 / 10	4 / 10		
	Respiratory region, lumen, amorphous eosinophilic material (Score)	10 / 10				9 / 10	1 / 10				10 / 10				4 / 10	3 / 10	3 / 10		
	Respiratory region, lumen, necrotic cells (Score)	9 / 10			1 / 10	10 / 10					10 / 10				5 / 10	5 / 10			
	Respiratory region, lumen, plant material (Score)	10 / 10				10 / 10					10 / 10				9 / 10	1 / 10			
Nose level 2	Respiratory epithelium, degeneration (Score)	10 / 10				10 / 10					10 / 10				4 / 10	1 / 10	3 / 10	2 / 10	
	Respiratory epithelium, hyperplasia (Score)	10 / 10				10 / 10					10 / 10				1 / 10	2 / 10	5 / 10	2 / 10	
	Respiratory epithelium, intraepithelial eosinophilic deposits (Score)	10 / 10				10 / 10					7 / 10	1 / 10	2 / 10		10 / 10				
	Respiratory epithelium, squamous epithelial metaplasia (Score)	10 / 10				10 / 10					10 / 10				2 / 10	1 / 10	6 / 10	1 / 10	
	Respiratory epithelium, ulceration (Score)	10 / 10				10 / 10					10 / 10				4 / 10	2 / 10	3 / 10	1 / 10	
	Respiratory region, lumen, amorphous eosinophilic material (Score)	10 / 10				10 / 10					10 / 10				5 / 10	2 / 10	3 / 10		
	Respiratory region, lumen, necrotic cells (Score)	9 / 10			1 / 10	10 / 10					10 / 10				9 / 10	1 / 10			
	Respiratory region, lumen, plant material (Score)	9 / 10			1 / 10	10 / 10					10 / 10				9 / 10	1 / 10			
	Olfactory epithelium, atrophy (Score)	9 / 10		1 / 10		7 / 10	1 / 10	1 / 10		1 / 10	10 / 10				3 / 10		1 / 10	4 / 10	2 / 10
	Olfactory epithelium, intraepithelial eosinophilic deposits (Score)	10 / 10				9 / 10			1 / 10		10 / 10				10 / 10				
	Olfactory epithelium, lamina propria, loss of nerve bundles (Score)	10 / 10				9 / 10			1 / 10		10 / 10				4 / 10		2 / 10		2 / 10
	Olfactory epithelium, squamous epithelial metaplasia (Score)	10 / 10				9 / 10		1 / 10			10 / 10				9 / 10		1 / 10		
	Olfactory region, lumen, amorphous eosinophilic material (Score)	10 / 10				10 / 10					10 / 10				9 / 10	1 / 10			
	Respiratory region, submucosal gland, ectasis (Score)	10 / 10				10 / 10					9 / 10	1 / 10			10 / 10				
Nose level 4	Olfactory epithelium, atrophy (Score)	10 / 10				9 / 10	1 / 10				10 / 10				3 / 10		5 / 10	2 / 10	
	Olfactory epithelium, lamina propria, loss of nerve bundles (Score)	10 / 10				10 / 10					10 / 10				9 / 10		1 / 10		
	Olfactory region, lumen, amorphous eosinophilic material (Score)	10 / 10				9 / 10	1 / 10				10 / 10				3 / 10		1 / 10	2 / 10	4 / 10
	Olfactory region, lumen, red blood cells (Score)	9 / 10	1 / 10			10 / 10					10 / 10				10 / 10				
Left lung	Alveolar lumen, hemorrhage (Score)	10 / 10				10 / 10					10 / 10				10 / 10				
	Alveolar lumen, lymphocytes/plasma cells (Score)	10 / 10				4 / 10	6 / 10				10 / 10				1 / 10	9 / 10			
	Alveolar lumen, neutrophilic granulocytes (Score)	10 / 10				4 / 10	6 / 10				10 / 10				1 / 10	9 / 10			
	Alveolar lumen, pigmented macrophage nests (Score)	10 / 10				7 / 10	3 / 10				10 / 10				8 / 10	2 / 10			
	Alveolar lumen, transudate/exudate (Score)	9 / 10	1 / 10			6 / 10	4 / 10				10 / 10				1 / 10	8 / 10	1 / 10		
	Alveolar lumen, unpigmented macrophages (Score)	8 / 10	2 / 10				1 / 10	6 / 10	3 / 10		9 / 10	1 / 10				1 / 10	2 / 10	7 / 10	
	Alveolar lumen, yellow pigmented macrophages (Score)	10 / 10				2 / 10	2 / 10	6 / 10			10 / 10				1 / 10		6 / 10	3 / 10	
	Perivascular mono-nuclear inflammatory cells (Score)	9 / 10	1 / 10			4 / 10	4 / 10	2 / 10			9 / 10	1 / 10			2 / 10	4 / 10	4 / 10		
	Thickened alveolar interstitium (Score)	10 / 10				7 / 10		3 / 10			10 / 10				9 / 10		1 / 10		

1 – 10
Number of animals per score (Incidence)

Values are incidences. Darker colors indicate higher incidences. 3R4F, reference cigarette; WBEC, whole-body exposure chamber; NOEC, nose-only exposure chamber.
