# Supplementary material for: Impact of whole‐body versus nose‐only inhalation exposure systems on systemic, respiratory, and cardiovascular endpoints in a 2‐month cigarette smoke exposure study in the ApoE−/− mouse model
Source: J Appl Toxicol. 2021 Apr 6;41(10):1598–619. doi: 10.1002/jat.4149 (PMC8519037; doi:10.1002/jat.4149)
Supplement: Supplementary file 8 — Table S4. Inflammatory mediators in BALF. [file JAT-41-1598-s001.pdf]

Supplementary Table 4. Inflammatory mediators in BALF.

		Sham WBEC	3R4F WBEC	Sham NOEC	3R4F NOEC
pg/mL	CXCL3	**15.76** ± 2.31	**179.84** ± 19.49 &	**16.37** ± 6.01	**114.12** ± 19.64 $&
	CCL2	**10.38** ± 2.63	**150.26** ± 44.67 &	**12.05** ± 4.17	**65.68** ± 8.26 &
	IL9	**41.31** ± 10.49	**55.98** ± 8.10	**92.80** ± 17.24 #	**59.41** ± 7.18
	CCL3	**17.63** ± 3.39	**45.71** ± 9.49 &	**13.69** ± 3.68	**56.25** ± 3.74 &
	CCL4	**5.95** ± 0.00	**65.23** ± 15.76 &	**8.73** ± 2.78	**55.02** ± 7.26 &
	CSF3	**3.48** ± 0.40	**33.77** ± 3.92 &	**5.83** ± 2.64	**50.19** ± 6.91 &
	CXCL10	**7.42** ± 0.89	**32.57** ± 6.57 &	**8.39** ± 2.25	**37.50** ± 5.44 &
	CXCL2	**31.09** ± 4.04	**39.54** ± 4.04	**21.38** ± 1.75	**29.79** ± 3.12 &
	IL6	**1.98** ± 0.78	**16.74** ± 4.79 &	**2.87** ± 1.20	**8.63** ± 1.63 &
	CSF2	**6.29** ± 0.56	**8.45** ± 1.42	**6.01** ± 0.56	**7.52** ± 1.13
	IL1A	**12.31** ± 4.17	**8.97** ± 2.98	**18.44** ± 5.07	**6.33** ± 0.80 &
	IL5	**3.99** ± 1.53	**5.70** ± 1.80	**3.02** ± 1.21	**4.95** ± 1.72
	IL13	**3.90** ± 0.00	**3.90** ± 0.00	**3.90** ± 0.00	**3.90** ± 0.00
	IL15	**3.70** ± 0.00	**3.70** ± 0.00	**3.70** ± 0.00	**3.70** ± 0.00
	IL1B	**3.94** ± 0.90	**4.57** ± 0.95	**2.90** ± 0.20	**3.09** ± 0.26
	IL12A+IL12B	**2.81** ± 0.28	**2.40** ± 0.00	**2.61** ± 0.21	**2.58** ± 0.18
	TNF	**1.15** ± 0.00	**2.94** ± 0.54 &	**1.15** ± 0.00	**2.46** ± 0.37 &
	IL12A	**2.38** ± 0.43	**1.95** ± 0.00	**1.95** ± 0.00	**2.32** ± 0.19
	IL10	**3.30** ± 1.09	**2.62** ± 0.76	**4.65** ± 0.88	**1.45** ± 0.23 &
	IL2	**2.11** ± 0.46	**1.88** ± 0.47	**2.48** ± 0.52	**1.37** ± 0.26
	CCL5	**1.35** ± 0.00	**1.35** ± 0.00	**1.35** ± 0.00	**1.35** ± 0.00
	IL17A	**0.25** ± 0.00	**0.27** ± 0.02	**0.25** ± 0.00	**1.01** ± 0.25 $&
	IL7	**0.89** ± 0.10	**0.78** ± 0.08	**0.79** ± 0.09	**0.74** ± 0.04
	IFNG	**0.55** ± 0.00	**0.55** ± 0.00	**0.60** ± 0.05	**0.55** ± 0.00
	IL4	**0.30** ± 0.06	**0.45** ± 0.10	**0.23** ± 0.02	**0.31** ± 0.07
ng/mL	SICAM1	**6.40** ± 0.56	**13.53** ± 1.55 &	**6.63** ± 0.48	**10.32** ± 0.86 &
	MMP9	**0.01** ± 0.00	**2.04** ± 0.31 &	**0.12** ± 0.10	**2.06** ± 0.19 &
	SERPIN1	**0.40** ± 0.02	**1.19** ± 0.06 &	**0.44** ± 0.05	**1.12** ± 0.10 &
	THBD	**0.57** ± 0.02	**1.27** ± 0.13 &	**0.82** ± 0.06 #	**1.05** ± 0.10
	PECAM1	**0.23** ± 0.02	**0.35** ± 0.04 &	**0.22** ± 0.02	**0.26** ± 0.02
	SELP	**0.18** ± 0.00	**0.18** ± 0.00	**0.18** ± 0.00	**0.18** ± 0.00
	SELE	**0.02** ± 0.00	**0.02** ± 0.00	**0.02** ± 0.00	**0.02** ± 0.00

Rank of Mean

1 4

Values were ranked over the four groups for each inflammatory mediator. The colors mark the group from the lowest to the highest average mean for each mediator, with a darker color indicating a higher value. Few subtle differences were noted between the WBEC and NOEC groups: Interleukin (IL) 17A, a pro-inflammatory cytokine, was significantly increased by NO exposure to CS but unaffected by WB exposure to CS. C-X-C motif ligand 3 (CXCL3), a chemokine, was increased upon CS exposure in both exposure chambers but significantly higher in the CS WBEC group than in the CS NOEC group. CCL, Chemokine (C-C motif) ligand; CSF, Colony-stimulating factor; CXCL, C-X-C motif ligand; IFNG, Interferon gamma; IL, Interleukin; NOEC, nose-only exposure chamber; MMP, Matrix metalloproteinase; PECAM, Platelet endothelial cell adhesion molecule; SELE, Selectin E; SELP, selectin P; SERPIN, serine protease inhibitor; SICAM, soluble Intercellular adhesion molecule; THBD, Thrombomodulin; TNF, Tumor necrosis factor; WBEC, whole-body exposure chamber.
